# Gut microbiota–immune crosstalk in osteoarthritis: pathogenic mechanisms and emerging therapeutic opportunities

**DOI:** 10.3389/fmicb.2026.1869199

**Published:** 2026-06-19

**Authors:** Tao Zhang, Bowen Tang, Zhitong Yu, Chuan Zhang, Zhangchao Pan, Chenxue Liang, Hongxin Zheng, Qingyan Wang

**Affiliations:** 1 Liaoning University of Traditional Chinese Medicine, Shenyang, China; 2 Second Affiliated Hospital, Liaoning University of Traditional Chinese Medicine, Shenyang, China; 3 First Affiliated Hospital, Liaoning University of Traditional Chinese Medicine, Shenyang, China

**Keywords:** chronic low-grade inflammation, gut microbiome, gut–joint axis, immune crosstalk, microbiome-based intervention, osteoarthritis

## Abstract

Osteoarthritis (OA) has traditionally been viewed as a degenerative joint disorder primarily associated with mechanical stress and progressive structural damage. Increasing evidence, however, indicates that immune imbalance and persistent low-grade inflammation are critically involved in disease initiation and progression. In this context, alterations in the gut microbiota have attracted growing attention due to their capacity to influence systemic immune responses. Here, we provide an integrated overview of the interactions between the gut microbiota and the immune system in OA and introduce the concept of a “gut microbiota–immune–joint axis” to describe this interconnected regulatory network. Disruption of the gut microbial ecosystem may impair intestinal barrier function and facilitate the entry of microbe-derived signals into the circulation. These signals subsequently activate inflammatory pathways, including TLR4/NF-κB and JAK/STAT cascades, leading to immune cell reprogramming, altered macrophage polarization, and imbalanced T-cell responses. The resulting chronic inflammatory state can extend beyond the intestine and contribute to pathological changes in synovial tissue, cartilage, and subchondral bone. In addition, we summarize current progress in microbiota-oriented therapeutic strategies, particularly the use of probiotics and prebiotics, and discuss their potential roles in modulating immune responses and restoring systemic homeostasis. Finally, we highlight existing challenges and propose future directions, emphasizing the importance of multi-omics integration and longitudinal clinical studies to better understand the dynamic nature of microbiota–immune interactions and to support the development of targeted interventions in OA.

## 1 Introduction

Osteoarthritis (OA) is one of the most prevalent degenerative joint disorders, characterized by structural alterations including cartilage degeneration, subchondral bone remodeling, and synovial inflammation. These changes ultimately lead to chronic pain, impaired joint function, and reduced quality of life (Wang et al., 2025; Hao et al., 2025). Traditionally, OA has been attributed to factors such as increased mechanical loading, cumulative joint wear, and age-related degeneration (Dang et al., 2025). However, these factors alone fail to fully explain the marked inter-individual variability and heterogeneity observed in disease progression. Increasing evidence suggests that OA is not merely a structural disorder but is also accompanied by persistent low-grade inflammation and immune dysregulation (Hu et al., 2025), indicating that it should be viewed, at least in part, as an immune–inflammatory disease.

The gut microbiota plays a critical role in maintaining immune homeostasis and regulating systemic inflammatory tone (Ding et al., 2025). In patients with OA, both the composition and function of the gut microbiota are altered, typically manifesting as reduced short-chain fatty acid (SCFA) production capacity, an increased abundance of potentially pro-inflammatory taxa, and a reshaping of microbial metabolic functions (de Sire et al., 2025; Gilat et al., 2025). These alterations can influence immune regulation through multiple mechanisms. For instance, microbe-associated molecular patterns such as lipopolysaccharide (LPS) can activate pro-inflammatory signaling pathways, including TLR4/NF-κB, thereby promoting cytokine release and elevating systemic inflammation (Gryaznova et al., 2024). In contrast, microbial metabolites such as SCFAs and tryptophan-derived compounds contribute to immune tolerance by modulating T-cell differentiation and macrophage polarization (Tran et al., 2025). In the context of OA, dysregulation of these microbiota-derived immune signals may drive a persistent state of low-grade systemic inflammation, which, through circulating inflammatory mediators, acts on synovial and cartilage tissues. This process promotes immune cell activation and extracellular matrix degradation, thereby accelerating disease progression (Jing et al., 2025; Edalat et al., 2024; Ayushman et al., 2025).

Although research on the gut microbiota and immune regulation in osteoarthritis (OA) has expanded substantially in recent years, and several reviews have summarized advances from perspectives such as gut microbiota-mediated regulation of age-related OA, the gut–joint axis, and inflammaging (Yu et al., 2025; Xi et al., 2025; Gaspar et al., 2025), with some studies additionally focusing on the role of microbiota-associated signals in immune inflammation (Zhang et al., 2026; Plewa et al., 2026), important gaps remain in the current understanding. Existing literature has largely concentrated on alterations in microbial composition, classical signaling mediators such as lipopolysaccharide (LPS) and short-chain fatty acids (SCFAs), or broad therapeutic implications. However, a systematic integration of how gut-derived signals further influence the OA-specific synovium–cartilage–subchondral bone microenvironment through immune modulation, as well as how these processes dynamically evolve during disease initiation and progression, is still lacking. Compared with previously proposed concepts such as the “gut–joint axis” or the “microbiota–immune–bone axis,” the present review places greater emphasis on the continuous interplay among microbial signals, immune homeostasis imbalance, and the local joint microenvironment in OA. Moreover, microbial structural components, metabolites, secreted factors, and nucleic acid-associated molecules are incorporated into a unified immunoregulatory framework and interpreted within the context of stage-specific disease evolution.

Based on this perspective, the present review systematically summarizes the immunomodulatory mechanisms mediated by microbial structural components, metabolites, secreted factors, and nucleic acid-associated molecules in the context of OA-related gut microbial dysbiosis and immune homeostasis disruption. In addition, the dynamic alterations of these mechanisms during both the initiation and progression stages of OA are comprehensively discussed. Finally, current advances in microbiota-related therapeutic strategies for OA are summarized, with the aim of providing new mechanistic insights into disease pathogenesis and identifying potential therapeutic targets from an integrated immunological perspective.

## 2 Gut microecological alterations and immune homeostasis in osteoarthritis

In recent years, advances in high-throughput sequencing and multi-omics approaches have enabled a more systematic characterization of gut microecological alterations associated with OA (Wang et al., 2026). Compared with healthy individuals, patients with OA generally exhibit remodeling of both the composition and functional capacity of the gut microbiota. However, specific taxonomic changes display substantial heterogeneity across studies (Wang et al., 2026; Wang et al., 2025). The direction and magnitude of microbial shifts are not always consistent, likely reflecting differences in host metabolic status, disease stage, dietary patterns, and medication use. Consequently, taxonomic profiling alone is insufficient to fully explain the role of the microbiota in OA, and current research increasingly emphasizes functional and microenvironmental perspectives.

At the ecological level, OA-associated microbial alterations are often accompanied by remodeling of the intestinal luminal microenvironment. Evidence suggests that an increased abundance of mucin-degrading bacteria, along with shifts in microbial substrate utilization, may influence the thickness and composition of the mucus layer (Shen et al., 2023; Arias et al., 2025). In parallel, changes in local oxygen tension and metabolite distribution can disrupt the balance between obligate anaerobes and facultative anaerobes, thereby altering the spatial organization of microbial communities (Yang et al., 2024; Narla et al., 2025). Such niche-level changes not only affect microbial stability but also modify the modes of interaction between microbes and the host, potentially facilitating sustained immune exposure to microbial-derived signals.

At the barrier level, ecological shifts in the microbiota can directly compromise intestinal epithelial integrity. Reduced expression of tight junction proteins and alterations in mucus layer structure increase intestinal permeability, allowing luminal microbe-associated molecules to translocate into the submucosa and systemic circulation (Yang et al., 2025; Segui-Perez et al., 2024). Importantly, this impairment does not necessarily manifest as overt inflammation but is more commonly associated with a state of low-grade, chronic immune stimulation (Saha et al., 2025). In the context of OA, such systemic immune activation may influence synovial tissues via circulating inflammatory mediators and immune cell trafficking, thereby reshaping the local immune microenvironment and increasing the susceptibility of cartilage to inflammatory damage (Yuan et al., 2025; Lönsjö et al., 2025).

At the immune level, intestinal immune homeostasis is also dynamically altered. Under physiological conditions, the mucosal immune system maintains selective tolerance to commensal microbes through mechanisms such as immunoglobulin A (IgA) secretion, immune cell tolerance, and antimicrobial peptide regulation (Carreto-Binaghi et al., 2024). In OA-associated states, alterations in IgA coating patterns and immune exclusion mechanisms may occur, affecting microbial spatial distribution and colonization stability (Wang, 2025; Panichi et al., 2024). Concurrently, the functional states of macrophages and T cells may shift toward enhanced pro-inflammatory responses and reduced immunoregulatory capacity (Arneth, 2026; Xi et al., 2025; Yang et al., 2025). These changes at the immunological level not only reflect a reconfiguration of host–microbiota interactions but also provide a basis for the persistence of systemic inflammation.

## 3 Molecular mechanisms and signaling regulation of gut microbiota–immune interactions

The gut microbiota modulates immune system function through multiple classes of signals, including structural components, metabolic products, and secreted factors (Sharma et al., 2025). These signals exhibit a degree of functional stratification. Microbial structural components are primarily recognized by pattern recognition receptors (PRRs), thereby initiating innate immune responses and inflammatory signaling. In contrast, microbial metabolites influence the direction and magnitude of immune responses by regulating immune cell differentiation and intracellular signaling pathway activity. Secreted factors and nucleic acid–associated molecules further contribute to the amplification and distal transmission of immune signals (Feng et al., 2025).

Concurrently, the immune system exerts reciprocal regulation over the microbiota through mechanisms such as PRR-mediated sensing, cytokine signaling, and immune cell recruitment, thereby shaping microbial composition and spatial organization and forming a dynamic, bidirectional interaction network (Keshavarz et al., 2025).

**Figure 1:**
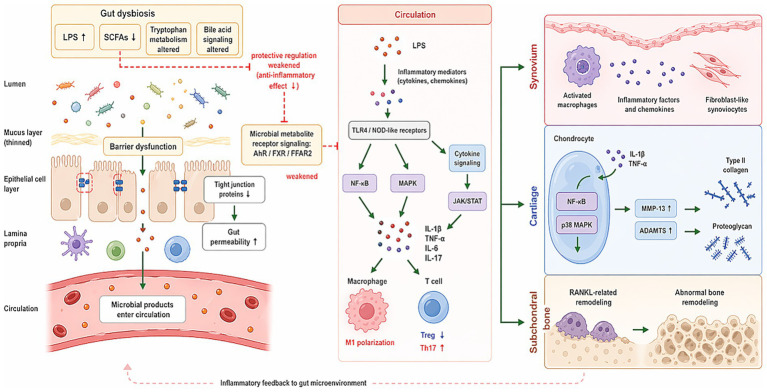
Mechanistic framework of the gut microbiota–immune–joint axis in osteoarthritis. Gut microbiota dysbiosis impairs intestinal barrier function and promotes the translocation of microbial products into the circulation, activating immune signaling pathways such as TLR4/NF-κB and JAK/STAT. This leads to macrophage activation, T cell imbalance, and low-grade systemic inflammation, which subsequently drive synovial inflammation, cartilage degradation, and subchondral bone remodeling. A feedback loop between joint inflammation and gut microenvironment may further exacerbate disease progression.

In the context of OA, this interplay extends beyond the intestinal microenvironment. Through low-grade systemic inflammation and the dissemination of immune signals, microbiota-derived effects can influence synovial and cartilage tissues, modulating macrophage polarization, inflammatory mediator expression, and chondrocyte catabolic activity (Jan et al., 2025; Gaspar et al., 2025) (see Figure 1 for an overview of the related mechanisms). When this balance is disrupted, it may give rise to a persistent low-grade inflammatory milieu, which, through a continuous interplay among immune dysregulation, metabolic disturbance, and structural damage, ultimately drives OA progression.

### 3.1 Molecular mechanisms by which the gut microbiota regulates immunity

#### 3.1.1 Immune regulation mediated by microbial structural components

Microbial structural components constitute a primary source of signals recognized by the host immune system, including lipopolysaccharide (LPS), lipoteichoic acid (LTA), and flagellin (Lin et al., 2026). These molecules are continuously sensed by pattern recognition receptors (PRRs), thereby modulating immune responses and maintaining immune surveillance (Seo and Lim, 2025). In the context of osteoarthritis (OA), microbial dysbiosis is often accompanied by an increased burden of pro-inflammatory molecules such as LPS (Piva et al., 2023). LPS can activate the NF-κB signaling pathway via Toll-like receptor 4 (TLR4), promoting the expression of pro-inflammatory cytokines and elevating systemic inflammatory tone (Wang and Xiao, 2023). This persistent low-grade inflammatory state can act on synovial tissues through circulating mediators, leading to immune cell activation and exacerbation of local inflammation (Hutton et al., 2025).

Beyond LPS, microbial structural proteins can also influence immune regulation through mechanisms such as molecular mimicry (Liu et al., 2025). Certain bacterial flagellins share structural similarities with host proteins, potentially inducing aberrant T-cell activation and enhancing the production of pro-inflammatory cytokines (Pang et al., 2025). In OA, this process may contribute to heightened synovial immune responses and indirectly modulate inflammatory signaling in chondrocytes (Kang et al., 2025). Additionally, surface adhesion factors expressed by pathogenic bacteria can influence macrophage polarization, favoring pro-inflammatory phenotypes and thereby altering the joint microenvironment (Pham and Monack, 2023).

Conversely, surface polysaccharides derived from commensal bacteria may exert immunomodulatory effects by promoting macrophage polarization toward anti-inflammatory phenotypes and enhancing tissue repair–associated responses (Guo et al., 2026). Such regulatory mechanisms are essential for maintaining immune homeostasis and limiting excessive inflammation. In the context of OA, dysregulation of structural component–mediated immune signaling may represent a critical link between microbial alterations and joint inflammation (Moulin et al., 2025).

#### 3.1.2 Immune regulation mediated by microbial metabolites

In contrast to microbial structural components, which primarily initiate inflammatory responses, microbial metabolites play a more decisive role in shaping the direction and magnitude of immune responses (Hou et al., 2025). These metabolites include SCFAs, secondary bile acids, tryptophan-derived metabolites, and other small signaling molecules (Qiu et al., 2025). Many of these compounds can cross the intestinal barrier and enter systemic circulation, where they interact with host receptors or modulate intracellular signaling pathways to influence immune cell activation and differentiation (Chen et al., 2024; Fiorucci et al., 2024).

Importantly, these metabolic signals exert effects not only within the intestinal immune environment but also at distal sites. In the context of OA, microbiota-derived metabolites can modulate synovial and cartilage biology through systemic inflammatory and immunoregulatory pathways, thereby contributing to disease progression (see Table 1).

**Table 1:** Immunomodulatory effects of microbiota-derived metabolites and their functional roles in OA.

Metabolite class	Representative molecule	Mechanism of action	OA-related effects	References
SCFAs	Butyrate	Inhibits HDAC activity, promotes Treg differentiation, and suppresses pro-inflammatory macrophage responses	Attenuates synovial inflammation and suppresses cartilage MMP expression	Parada-Venegas et al. (2025), Ning et al. (2025)
SCFAs	Acetate	Enhances neutrophil chemotaxis and promotes Th17 responses via FFAR2	Promotes a pro-inflammatory microenvironment under specific conditions	Lv et al. (2025), Wang et al. (2026)
SCFAs	Valerate	Inhibits HDAC activity and upregulates Foxp3 expression, enhancing immune tolerance	Suppresses inflammation and delays cartilage degradation	Liang et al. (2025), Liu et al. (2025), Kumari et al. (2024)
SCFAs	Isobutyrate/Isovalerate	Activates FFAR2/FFAR3 to regulate immune cell chemotaxis and inflammatory intensity	Contributes to the regulation of local inflammatory balance	Mårtensson et al. (2023), Jevtić et al. (2025), Ren et al. (2026)
Bile acids	Secondary bile acids	Activate FXR/TGR5 to suppress inflammatory cytokine expression and modulate immune cell function	Regulate synovial inflammatory responses	Song et al. (2025), Yang et al. (2025)
Bile acids	Lithocholic acid	Inhibits Th17 differentiation and enhances Treg function via VDR	Improves immune homeostasis and reduces inflammation	Zhang et al. (2024), Bao-Anh et al. (2025)
Tryptophan metabolites	Indole derivatives	Regulate dendritic cell and T-cell differentiation via AhR signaling	Modulate inflammatory responses and immune balance	Helm and Zhou (2023), Sadeghi Shermeh et al. (2023)
Nucleotides	ATP	Promotes pro-inflammatory cytokine expression and enhances Th17 responses	Exacerbates synovial inflammation	Hamoudi et al. (2026), Ma et al. (2025)
Cyclic nucleotides	c-di-AMP	Activates the STING pathway to induce interferon responses and regulate innate immunity	Influences the joint immune microenvironment	Deng et al. (2025), He et al. (2024), Zhang et al. (2025)

SCFAs are among the most representative microbial metabolites, and their immunomodulatory effects are highly context-dependent (Wang et al., 2024). Butyrate and valerate can promote the differentiation of regulatory T cells (Tregs) and suppress pro-inflammatory macrophage responses by inhibiting histone deacetylase (HDAC) activity, thereby reducing overall inflammatory levels (Saadh et al., 2025). This process contributes to the attenuation of synovial inflammation and the downregulation of molecules involved in cartilage matrix degradation. Meanwhile, certain short-chain fatty acids may also regulate neutrophil chemotaxis and Th17-associated immune responses through receptors such as FFAR2 (Huang et al., 2025). It should be noted that direct evidence supporting these mechanisms is currently derived primarily from studies of rheumatoid arthritis and other inflammatory disorders, and their precise roles in OA remain to be further elucidated. Nevertheless, emerging OA-related studies have suggested that alterations in short-chain fatty acid levels may be associated with synovial inflammation and abnormal cartilage metabolism (Ning et al., 2025; Wang et al., 2026).

Bile acid metabolites also play a role in immune regulation, primarily through receptors such as FXR, TGR5, and VDR (Xia et al., 2025). Certain secondary bile acids can suppress the immunostimulatory capacity of dendritic cells and promote the expansion of regulatory T cells, thereby dampening inflammatory responses (Oleszycka et al., 2023). Others inhibit Th17 cell differentiation and reduce the expression of inflammation-associated transcription factors, thus limiting the propagation of inflammation (Shao-Yu et al., 2025). In the context of OA, these effects may help modulate synovial immune responses and influence subchondral bone remodeling. Notably, the immunological effects of bile acids are both concentration- and microenvironment-dependent, and their functional outcomes may shift with changes in microbial composition and metabolic status (Mohanty et al., 2024).

In addition, various small-molecule signaling mediators derived from the microbiota can participate in immune regulation through specific pathways (Fioretto et al., 2025). Microbial-derived ATP can promote the expression of pro-inflammatory cytokines and enhance Th17 responses, thereby amplifying inflammatory signaling (Tian et al., 2025). Cyclic dinucleotides, such as cyclic di-AMP, can activate the STING pathway to induce interferon production and modulate innate immune responses (Chen et al., 2023). Tryptophan-derived metabolites regulate dendritic cell and T-cell function via activation of the aryl hydrocarbon receptor (AhR), contributing to the maintenance of immune homeostasis (Solvay et al., 2023). In OA, these metabolic signals may influence disease progression by modulating the synovial immune microenvironment and inflammatory responses in chondrocytes, thereby either exacerbating or alleviating pathological processes.

#### 3.1.3 Immunological effects of microbial secreted factors and nucleic acid–associated molecules

In addition to structural components and metabolic products, the gut microbiota can regulate host immunity through the secretion of diverse functional molecules (Li and Guo, 2026). These include outer membrane vesicles (OMVs), secreted proteins and toxins, extracellular nucleic acids, and quorum-sensing signals (Luo et al., 2024). Unlike soluble metabolites, these signaling molecules often exhibit greater stability and delivery capacity, enabling them to traverse the intestinal barrier and be taken up by immune cells, thereby exerting regulatory effects at distal sites (Abubakari et al., 2025; Pilapitiya et al., 2025). In the context of OA, such microbiota-derived signals may circulate systemically and contribute to the modulation of synovial inflammation and the joint microenvironment (Zhang et al., 2026).

Outer membrane vesicles are nanoscale structures actively released by Gram-negative bacteria and contain diverse bioactive components, including proteins, lipids, and nucleic acids (Wang et al., 2025). Previous studies have shown that outer membrane vesicles can cross the epithelial barrier and be internalized by macrophages and dendritic cells. Their cargo can activate inflammatory pathways such as NF-κB and STAT3, thereby inducing pro-inflammatory factor expression and modulating immune responses (Zheng et al., 2024). It should be noted that current research on outer membrane vesicles has mainly focused on periodontitis, intestinal inflammation, and cancer, while direct evidence in OA remains relatively limited. Nevertheless, related studies suggest that microbiota-derived vesicles may contribute to OA-associated alterations in the joint microenvironment through systemic inflammation and synovial immune activation (Niu et al., 2025; Wang et al., 2025).

Microbial secreted proteins and toxins also play significant roles in immune regulation (Lata et al., 2026). Certain bacterial proteins can disrupt epithelial tight junctions, increasing intestinal permeability and facilitating the translocation of microbe-associated signals into systemic circulation (Nie et al., 2024; Zhao and Lin, 2025). In parallel, these molecules can directly activate immune cells by engaging inflammatory signaling pathways such as NF-κB and STAT3, promoting Th17 responses and amplifying inflammatory cascades (Zhang et al., 2025). In OA, such mechanisms may elevate systemic inflammatory levels, sustain synovial inflammation, and contribute to the activation of cartilage-degrading pathways (Zhang et al., 2025). Conversely, some commensal bacteria secrete bioactive peptides with immunosuppressive properties, which can inhibit dendritic cell maturation and attenuate inflammatory responses, thereby contributing to the maintenance of immune homeostasis (Weaver, 2024).

Under conditions of chronic inflammation or microbial dysbiosis, the gut microbiota may also release extracellular DNA and RNA fragments (Guo et al., 2025). These nucleic acid molecules can be recognized by pattern recognition receptors, activating pathways such as TLRs and the cGAS–STING axis, leading to the production of type I interferons and other inflammatory mediators that enhance innate immune responses (Guo et al., 2025). This process not only influences local intestinal immunity but may also exert systemic effects on joint tissues, altering synovial immune status and impacting chondrocyte function. In addition, quorum-sensing molecules can modulate immune responses through interactions with host receptors, with effects that are highly dose-dependent (Duan et al., 2023). At physiological levels, these signals help maintain barrier integrity and immune balance, whereas excessive accumulation may induce oxidative stress and inflammatory responses (Gates et al., 2026).

Overall, microbial secreted factors and nucleic acid–associated molecules provide an additional layer of communication between the microbiota and the immune system. Under conditions of dysbiosis or impaired barrier function, this regulatory network may shift toward persistent inflammatory stimulation, allowing gut-derived immune signals to exert sustained systemic effects on synovial and cartilage tissues, thereby promoting chronic inflammation and accelerating the progression of joint structural damage.

### 3.2 Immune regulation of gut microbiota composition and spatial organization

The immune system not only responds to signals derived from the gut microbiota but also actively regulates microbial composition and spatial distribution (Álvarez Calatayud et al., 2025). Under physiological conditions, the host immune system distinguishes between commensal microbes and potential pathogens, maintaining tolerance toward the former while rapidly initiating clearance mechanisms against the latter (Maciel-Fiuza et al., 2023). This selective regulation relies on the coordinated actions of innate immune recognition, effector molecule release, and adaptive immune responses, thereby preserving the stability of the intestinal microecosystem (Shao et al., 2023).

At the level of innate immunity, macrophages and neutrophils restrict excessive bacterial growth and prevent microbial translocation through the production of ROS and antimicrobial factors (Pradeep et al., 2024). Under inflammatory conditions, these effector molecules can significantly alter the intestinal microenvironment, including oxygen tension, nutrient availability, and local metabolic conditions. Such changes may selectively promote the expansion of facultative anaerobes while suppressing obligate anaerobes (Mansoori Moghadam et al., 2025), thereby contributing to microbial dysbiosis. In the context of OA, systemic inflammatory states may continuously reshape the nature and burden of microbiota-derived signals, further amplifying systemic inflammation and promoting synovial immune activation and joint microenvironmental imbalance (Sun et al., 2025).

At the mucosal immune level, secretory IgA plays a central role in regulating microbial spatial organization (Zeng et al., 2025). IgA selectively coats bacteria, limiting their direct contact with the epithelial surface and modulating their colonization capacity and metabolic activity (Zhang et al., 2023). This mechanism is essential for maintaining a stable host–microbiota interface. Disruption of IgA-mediated regulation may allow certain bacterial populations to gain a colonization advantage, thereby disturbing the existing ecological balance (Zhao et al., 2025). In OA-associated conditions, shifts in immune homeostasis may impair IgA-mediated microbial regulation, indirectly altering microbial functional profiles (Lisicka et al., 2025).

In addition, antimicrobial peptides secreted by intestinal epithelial cells and Paneth cells are critical for maintaining microbial homeostasis (Okumura and Takeda, 2024). These peptides can directly inhibit bacterial growth or influence microbial spatial distribution, contributing to the establishment of a stable microenvironment (Pereira et al., 2026). During inflammation, both the expression levels and distribution patterns of antimicrobial peptides may be altered, thereby affecting microbial composition (Svensson and Nilsson, 2025). Importantly, these changes are not confined to the intestinal milieu but may also influence systemic immune status by modulating the production of microbiota-derived metabolites.

### 3.3 Interplay among the gut microbiota, immunity, and osteoarthritis

The interaction between the gut microbiota and the immune system may contribute to the development of osteoarthritis (OA) through alterations in barrier function and the systemic dissemination of microbial-derived signals (Richie and Lee, 2025). Under conditions of microbial dysbiosis, reduced expression of intestinal epithelial tight junction proteins and disruption of the mucus layer can increase intestinal permeability, allowing lipopolysaccharide (LPS) and other microbiota-associated molecules to enter the circulation (Perrin et al., 2025). These molecules can activate NF-κB and MAPK signaling pathways through Toll-like receptor 4 (TLR4) and NOD-like receptors, thereby inducing the expression of inflammatory mediators such as IL-1β, TNF-α, and IL-6, which are associated with persistent low-grade systemic inflammation (Sawoo and Bishayi, 2024). In parallel, alterations in tryptophan metabolism and bile acid signaling may regulate immune cell differentiation through the aryl hydrocarbon receptor (AhR) and farnesoid X receptor (FXR) pathways, shifting immune responses toward a pro-inflammatory phenotype (Zhao et al., 2026).

On this basis, gut-derived signals do not merely influence OA through generalized systemic inflammation, but may further act directly on the OA-specific synovium–cartilage–subchondral bone microenvironment. Macrophages and fibroblast-like synoviocytes within synovial tissue are highly sensitive to circulating inflammatory mediators and microbiota-associated molecules (Schonfeldova et al., 2025). Activation of TLRs and cytokine receptor signaling can promote polarization of synovial macrophages toward an M1-like pro-inflammatory phenotype and enhance the release of IL-1β, TNF-α, IL-6, and chemokines through the NF-κB and JAK/STAT pathways (Li et al., 2025). Meanwhile, fibroblast-like synoviocytes can further upregulate inflammatory cytokine and chemokine expression, thereby promoting the recruitment of neutrophils, monocytes, and T cells and establishing a local synovial inflammatory amplification network (Zec et al., 2023). Th17-associated signaling and IL-17 secretion may further intensify the inflammatory cascade and influence bone metabolism through the RANKL pathway (Wu et al., 2024).

A close interplay exists between synovial inflammation and chondrocyte catabolism. IL-1β, TNF-α, and IL-6 released by M1-like synovial macrophages and activated fibroblast-like synoviocytes can act on chondrocytes, activating the NF-κB and p38 MAPK pathways and subsequently upregulating matrix metalloproteinase-13 (MMP-13) and ADAMTS family proteins, thereby promoting degradation of type II collagen and proteoglycans (Lu et al., 2024). At the same time, the inflammatory microenvironment suppresses chondrocyte anabolic activity and induces apoptosis, further impairing cartilage repair capacity. Conversely, damage-associated molecular patterns and matrix degradation fragments released by injured chondrocytes can further stimulate synovial macrophages and fibroblast-like synoviocytes, perpetuating local inflammatory amplification. This bidirectional synovium–cartilage interaction enables gut-derived immune signals to be translated into persistent inflammation and matrix destruction within the local OA joint microenvironment.

At the level of subchondral bone, persistent inflammatory mediators can regulate osteoclast activity through the RANK/RANKL/OPG axis, leading to abnormal bone remodeling, altered joint biomechanics, and aggravated cartilage injury (Di Cicco et al., 2024; Sobacchi et al., 2025). In addition, inflammatory and metabolic alterations may, in turn, reshape the intestinal microenvironment and further influence microbial composition (He et al., 2026). Systemic inflammation can alter host metabolism and intestinal nutrient availability while simultaneously promoting the expansion of specific microbial populations through immune-mediated niche regulation, thereby exacerbating microbial dysbiosis (Zheng et al., 2026). Consequently, gut microbiota alterations, immune dysregulation, synovium–cartilage interactions, and subchondral bone remodeling may form a self-reinforcing positive feedback loop, driving OA progression from early immune imbalance to persistent structural degeneration.

## 4 Dynamic evolution of immune–microbiota interactions in the initiation and progression of OA

**Figure 2:**
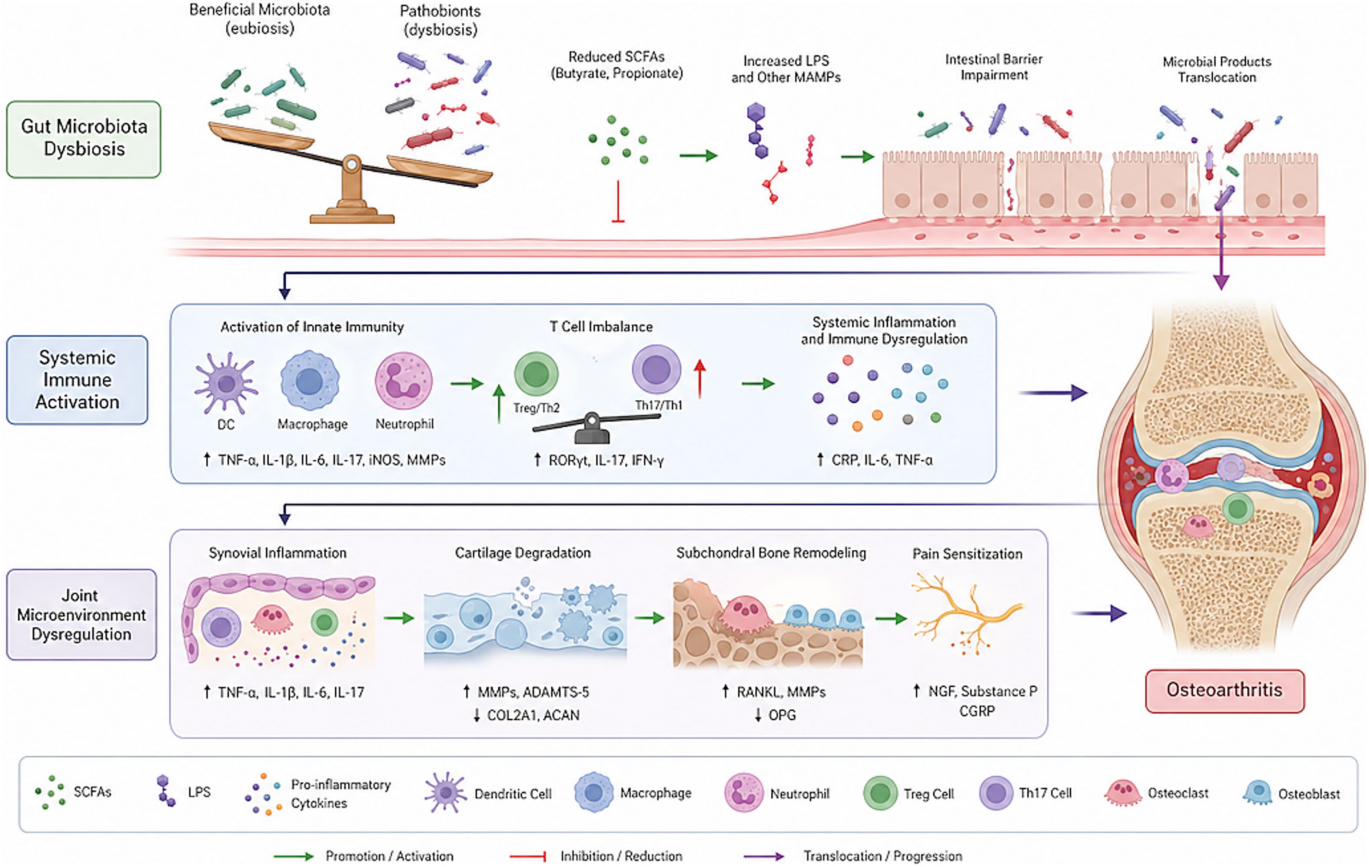
Conceptual model of the gut microbiota–immune–joint axis in osteoarthritis progression. Gut microbiota dysbiosis impairs intestinal barrier function and promotes the translocation of microbial products into the circulation, leading to immune activation and low-grade systemic inflammation. This immune dysregulation contributes to synovial inflammation, cartilage degradation, subchondral bone remodeling, and pain sensitization, ultimately driving osteoarthritis progression.

The interplay between the gut microbiota and the immune system exhibits distinct stage-specific characteristics during the progression of OA. From early immune dysregulation and impairment of barrier function to the sustained amplification of inflammatory responses and subsequent joint structural remodeling, microbiota–immune interactions are involved throughout the entire course of disease evolution (Meléndez-Oliva et al., 2025). Based on this dynamic process, OA-related microbiota–immune interactions can be broadly divided into an initiation phase and a progression phase, thereby providing a clearer framework for understanding their functional roles at different stages of the disease (see Figure 2).

### 4.1 Microbiota–immune interactions in the initiation phase of OA

#### 4.1.1 Low-grade inflammation and immune dysregulation

Under physiological conditions, the immune system maintains a dynamic balance of inflammatory responses through the recognition and tolerance of commensal microorganisms (Boem et al., 2025). Once microbial composition is disrupted, this balance becomes impaired, potentially leading to a persistent state of low-grade inflammation. In the context of microbial dysbiosis, levels of microbiota-associated molecules such as LPS increase and are more likely to enter the systemic circulation (Shaheen et al., 2025; Mohr et al., 2022), where they activate macrophages and monocytes through pathways including TLR4/NF-κB, thereby inducing the expression of inflammatory mediators such as IL-1β, TNF-α, and IL-6 (Xie et al., 2026). Meanwhile, microbial alterations can regulate macrophage polarization through signaling pathways such as STAT and MAPK, promoting their transition toward a pro-inflammatory phenotype and consequently amplifying inflammatory responses (Zhang et al., 2026). Concurrently, reductions in certain anti-inflammatory metabolites further weaken immunoregulatory capacity.

At the level of adaptive immunity, alterations in the gut microbiota and its metabolites can influence T-cell differentiation. Reduced short-chain fatty acid production and abnormal tryptophan metabolism may regulate Foxp3 expression through pathways such as FFAR2 and AhR, leading to a decline in regulatory T-cell populations while enhancing Th17 responses (Kanno et al., 2023; Endo et al., 2022), thereby shifting immune homeostasis toward a pro-inflammatory state (Akhter et al., 2023). This immune imbalance subsequently acts on joint tissues through circulating inflammatory mediators, inducing early activation of synovial macrophages and amplifying inflammatory responses via the NF-κB and JAK/STAT pathways (Wu et al., 2025; Wen and Liu, 2025). At the same time, chondrocytes exposed to IL-1β and TNF-α upregulate catabolism-related gene expression and exhibit reduced matrix synthesis capacity (Sha et al., 2022). Although overt structural damage has not yet occurred at this stage, the local immune microenvironment has already been altered, establishing the foundation for subsequent synovial inflammation and cartilage degeneration.

#### 4.1.2 Barrier dysfunction

Against the background of persistent low-grade inflammation, intestinal barrier integrity gradually becomes compromised (Selvakumar and Samsudin, 2025). Inflammatory mediators disrupt epithelial tight junction protein expression and interfere with epithelial cell renewal processes, thereby increasing intestinal permeability (Zammarchi et al., 2024). Simultaneously, alterations in microbial composition weaken microbiota-mediated support of the mucosal barrier, facilitating the translocation of microbiota-associated signals across the epithelium into the systemic circulation. Under dysbiotic conditions, the expansion of certain opportunistic pathogens may further damage epithelial structure through multiple mechanisms (Wang et al., 2024). For example, bacterial toxins and metabolites can reduce tight junction protein expression and impair epithelial integrity, whereas molecules such as LPS activate epithelial and immune cells through the TLR4/NF-κB pathway, thereby further amplifying local inflammatory responses (Li et al., 2023). Persistent inflammatory conditions can additionally suppress epithelial metabolism and renewal, impair barrier repair capacity, and ultimately establish a positive feedback loop characterized by progressively increased intestinal permeability (Yang et al., 2026).

Furthermore, dietary and metabolic status may indirectly regulate barrier integrity through microbiota-mediated mechanisms (Ma and Lee, 2025). Gut microbial alterations associated with high-fat diets can increase bile acid metabolite levels, and under certain conditions these molecules may impair epithelial cell function and exacerbate barrier damage through activation of inflammation-related signaling pathways (Cai et al., 2024). Meanwhile, reduced short-chain fatty acid production weakens epithelial energy supply and anti-inflammatory capacity, further compromising barrier stability (Andrani et al., 2024). Following barrier disruption, microbiota-associated molecules such as LPS and bacterial DNA are more likely to enter the circulation, thereby creating conditions that facilitate immune activation in distant tissues.

#### 4.1.3 Early alterations in the local joint immune microenvironment

On this basis, circulating inflammatory mediators and microbiota-associated molecules further act on joint tissues and are recognized by synovial macrophages (Holub et al., 2023). These signals activate NF-κB and JAK/STAT signaling pathways through TLR- and cytokine receptor-related mechanisms, inducing inflammatory mediator expression and promoting chemokine release, thereby driving the recruitment of monocytes and neutrophils into synovial tissue (Dreo et al., 2024; Haowen et al., 2025). Under persistent stimulation, synovial fibroblasts become actively involved in the local response by upregulating inflammation-related gene expression and cooperating with immune cells to sustain cytokine production, allowing inflammatory signaling to persist within the joint microenvironment (Tung et al., 2025).

Cartilage tissue is particularly sensitive to sustained low-level inflammation. IL-1β and TNF-α can induce chondrocytes to upregulate matrix-degrading enzymes while simultaneously suppressing the synthesis of type II collagen and proteoglycans, gradually shifting chondrocytes from an anabolic to a catabolic state (Su et al., 2025). In parallel, inflammatory signaling influences osteoclast activity within subchondral bone through RANKL-related pathways, disrupting bone metabolic homeostasis and altering joint biomechanics (Zhao et al., 2025), thereby further increasing mechanical stress on cartilage. Although substantial structural destruction is not yet evident at this stage, the synovium, cartilage, and subchondral bone have already entered a primed and vulnerable state that provides the basis for subsequent inflammatory amplification and progressive joint degeneration.

### 4.2 Microbiota–immune interactions in the progression phase of OA

#### 4.2.1 Sustained amplification of synovial inflammation

Persistent synovial inflammation is one of the major characteristics of OA progression (Mobasheri et al., 2026). Building upon the low-grade inflammatory state established during the initiation stage, inflammatory responses gradually shift from transient activation to a chronically sustained condition. This process is continuously amplified through interactions between local immune cells and joint tissues, ultimately resulting in the establishment of a stable inflammatory microenvironment (Deng et al., 2026). Among these mechanisms, persistent activation and functional remodeling of synovial immune cells represent critical events. Studies have demonstrated increased macrophage infiltration and sustained activation within synovial tissues from patients with OA (Del Sordo et al., 2023). Immunohistochemical and transcriptomic analyses further indicate persistent activation of the NF-κB and MAPK signaling pathways, accompanied by elevated expression of inflammatory mediators such as IL-1β, TNF-α, and IL-6 (Wang et al., 2023).

Fibroblast-like synoviocytes progressively participate in maintaining inflammation by secreting inflammatory cytokines and chemokines, including IL-6 and CCL2, thereby continuously recruiting immune cells and reinforcing local inflammatory responses (Zhang et al., 2024). At the same time, inflammatory mediators released by immune cells act reciprocally on synovial cells, further promoting their activation and establishing a stable intercellular inflammatory network (Zheng et al., 2025). In addition, microbiota-associated signals such as lipopolysaccharide (LPS) and bacteria-derived nucleic acids may continuously activate TLR and NOD signaling pathways, thereby enhancing NF-κB and JAK/STAT pathway activity and sustaining inflammatory cytokine expression (Eriksen et al., 2026; Stierschneider and Wiesner, 2023). During this process, microbiota-derived signals may provide persistent upstream input to the inflammatory network, which, together with intercellular positive feedback mechanisms, contributes to the chronic persistence of synovial inflammation.

#### 4.2.2 Cartilage degradation and subchondral bone remodeling

With the persistence of inflammatory responses, joint structural alterations gradually evolve from reversible metabolic dysregulation into stable pathological abnormalities. At the cartilage level, chronic inflammatory signaling disrupts the balance between matrix synthesis and degradation (Vonk, 2025), progressively impairing the anabolic function of chondrocytes while allowing catabolic processes to predominate (Zhang et al., 2026). As a result, cartilage progressively thins and loses its elastic properties. Meanwhile, within subchondral bone, the inflammatory microenvironment disrupts the coordination between bone resorption and bone formation, thereby inducing abnormal bone remodeling and altering joint biomechanics (Zapata-Linares et al., 2025).

As the disease progresses, the interaction between cartilage and subchondral bone becomes increasingly pronounced. Cartilage damage alters local mechanical stress distribution, whereas abnormalities in subchondral bone structure further increase mechanical stress on cartilage, thereby generating a structural amplification effect that promotes cumulative tissue damage (Pueyo Moliner et al., 2025). At this stage, inflammation no longer functions merely as an initiating factor but instead becomes a major driving force sustaining structural abnormalities. The persistent inflammatory environment prevents restoration of tissue homeostasis and promotes the transition of OA from localized structural changes to global joint functional imbalance.

In addition, OA-associated systemic inflammation and metabolic abnormalities may themselves exert reciprocal effects on the intestinal microenvironment. Chronic inflammatory conditions can impair intestinal epithelial barrier stability through inflammatory mediators and stress-related signaling pathways while simultaneously altering local intestinal oxidative stress and metabolic conditions, thereby influencing microbial composition and metabolic function. Furthermore, OA is frequently accompanied by obesity, insulin resistance, and metabolic syndrome, all of which may further reshape gut microbial ecology through alterations in dietary patterns, bile acid metabolism, and host nutrient availability.

## 5 Advances in microbiota-targeted interventions for OA

The interplay between the gut microbiota and the immune system has been increasingly recognized as a contributing factor in the pathogenesis of OA (Plewa et al., 2026). At present, registered randomized controlled trials in OA are mainly focused on probiotic and prebiotic interventions, whereas strategies aimed at direct microbiota reconstruction, such as fecal microbiota transplantation (FMT), still lack clinical evidence in OA. Notably, FMT has been discussed in other immune- and inflammation-related diseases as a potential therapeutic approach for correcting microbial dysbiosis and immune abnormalities, which may provide cross-disease insights for future exploration of microbiota reconstruction strategies in the context of OA (Fortuna et al., 2024; Chasov et al., 2024).

**Figure 3:**
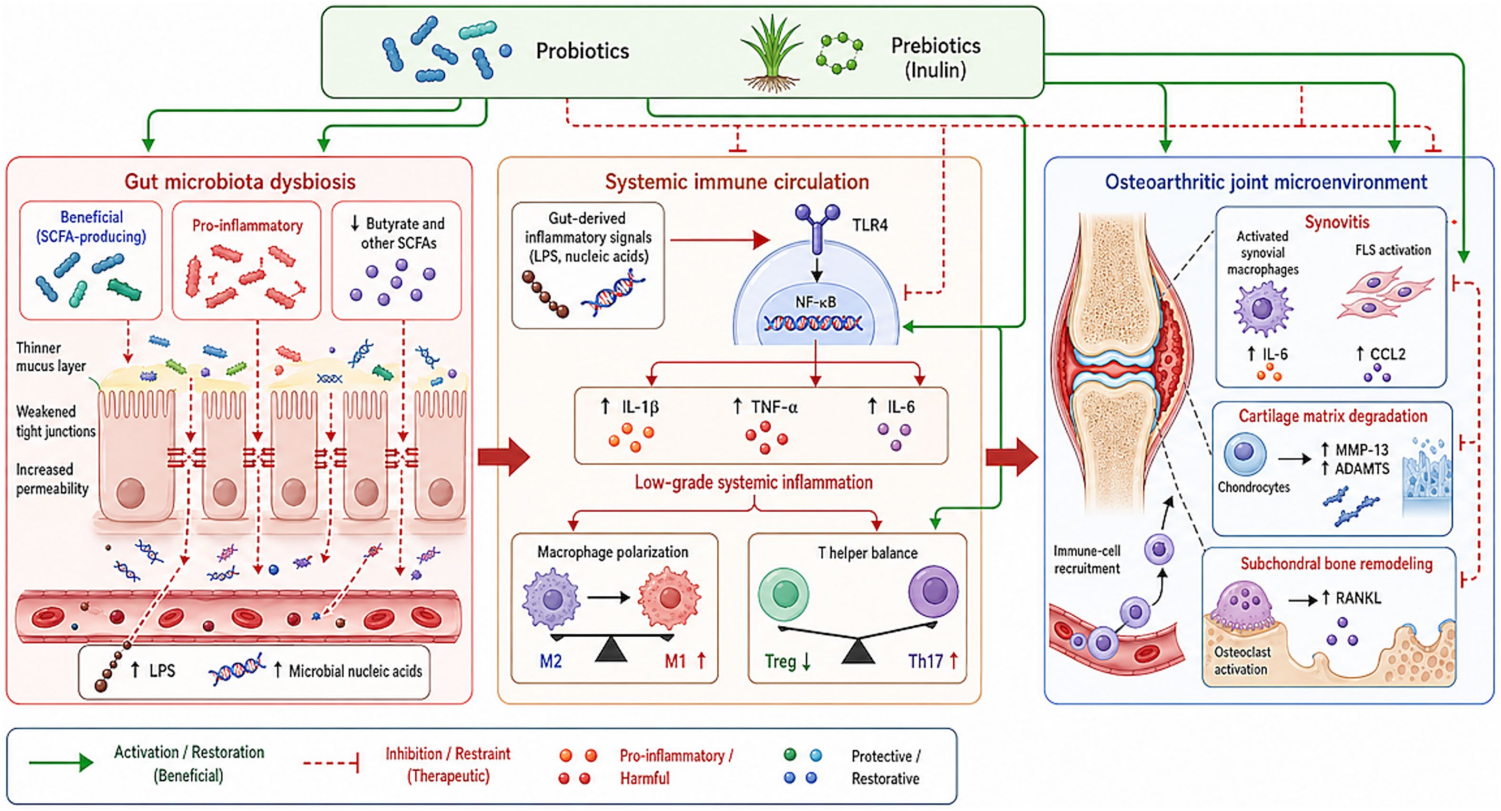
Microbiota-targeted therapeutic strategies in osteoarthritis: modulation of the gut–immune–joint axis. Probiotics and prebiotics modulate gut microbiota composition and metabolic activity, improving intestinal barrier integrity and reducing the translocation of microbial-derived inflammatory signals. This leads to attenuation of immune activation and low-grade systemic inflammation, thereby alleviating synovial inflammation, cartilage degradation, and subchondral bone remodeling in osteoarthritis.

Most existing studies have used pain relief and improvement in joint function as primary clinical endpoints, while gradually incorporating assessments of microbiota-related metabolic profiles and low-grade inflammatory status (see Table 2). The potential mechanistic pathways underlying these interventions, as well as their effects on the joint microenvironment, are illustrated in Figure 3.

**Table 2:** Clinical trials of microbiota-targeted interventions in OA.

Intervention type	NCT identifier	Phase	Sample size	Intervention	Primary outcomes
Probiotics	NCT04267432	Not applicable	102	TCI633 probiotics	Pain relief and functional improvement in osteoarthritis
Probiotics	NCT07364578	Phase IV	146	Probiotic formulation	Western Ontario and McMaster Universities Osteoarthritis Index (WOMAC)
Probiotics	NCT06459700	Not applicable	86	Probiotic supplements	Knee injury and Osteoarthritis Outcome Score (KOOS-12)
Prebiotics	NCT04172688	Not applicable	54	Oligofructose-enriched inulin	Changes in 30-s chair stand test, 40-meter fast-paced walk test, Timed Up and Go test, 6-min walk test, and knee function
Prebiotics	NCT07189585	Phase II	84	Inulin	Change in serum LPS concentration from baseline to 8 weeks

Current evidence suggests that probiotic supplementation can, to some extent, improve pain and joint function scores in patients with OA, as reflected by commonly used indices such as WOMAC and KOOS (NCT04267432, NCT07364578, NCT06459700) (Kouraki et al., 2026). These effects are not limited to alterations in microbial composition but may also involve modulation of immune responses. Studies indicate that probiotics can reduce circulating levels of LPS, thereby attenuating sustained activation of the TLR4/NF-κB signaling pathway and decreasing the production of pro-inflammatory cytokines such as IL-1β and TNF-α (Tian et al., 2025). In addition, probiotics may restore the balance between regulatory Tregs and Th17 cells by promoting SCFA production and regulating tryptophan metabolism, shifting immune responses from a pro-inflammatory state toward relative homeostasis (Kim, 2023). At the joint level, these immunomodulatory effects may manifest as reduced activation of synovial macrophages and diminished inflammatory signaling in chondrocytes, thereby slowing extracellular matrix degradation to some extent.

In contrast, prebiotics primarily exert their effects through modulation of microbial metabolism and intestinal barrier function. Clinical studies have shown that supplementation with compounds such as inulin not only improves functional outcomes but also reduces circulating LPS levels (NCT04172688, NCT07189585), suggesting a role in lowering metabolic endotoxin burden and associated inflammatory tone (Wang et al., 2026). Mechanistically, these effects are largely mediated by enhanced production of SCFAs. Metabolites such as butyrate serve as an energy source for epithelial cells and can suppress NF-κB signaling while promoting regulatory T-cell differentiation, thereby mitigating chronic inflammation (Chen et al., 2025). Furthermore, prebiotics improve intestinal barrier integrity, reducing the translocation of microbe-associated molecules into the circulation and consequently decreasing inflammatory stimulation of distal joint tissues (Acharya et al., 2024). In this way, metabolic regulation at the intestinal level may be translated into functional effects within the joint microenvironment.

## 6 Conclusions and future perspectives

Overall, current evidence suggests that gut microbiota-associated signals may contribute to the initiation and progression of OA through alterations in barrier integrity, disruption of immune homeostasis, and remodeling of the synovium–cartilage–subchondral bone microenvironment. In the present review, we further integrated the immunomodulatory effects of microbial structural components, metabolites, secreted factors, and nucleic acid-associated molecules, and systematically summarized the dynamic alterations of the “gut microbiota–immune–joint axis” across both the initiation and progression stages of the disease. Compared with the traditional view of OA as a purely mechanical degenerative disorder, accumulating evidence increasingly supports the concept that OA is also characterized by low-grade inflammation and immune dysregulation, with the gut microbiota potentially serving as a critical link connecting systemic inflammation and local joint pathology.

It should be emphasized that current microbiota-targeted intervention studies in OA still exhibit substantial limitations. Existing randomized controlled trials generally involve relatively small sample sizes, with most studies enrolling only several dozen to slightly more than one hundred participants. In addition, some investigations remain at an early exploratory stage, including trials classified as “Not Applicable,” and therefore lack rigorous control group designs and long-term follow-up data. Consequently, their clinical conclusions should be interpreted with caution. Moreover, considerable heterogeneity exists among studies with regard to strain selection, dosage, intervention duration, and patient metabolic background, further complicating interpretation of the available findings.

Furthermore, probiotic and prebiotic interventions are currently often discussed as broad, generalized concepts, despite the possibility that different microbial strains and metabolic substrates exert distinct immunomodulatory effects. For instance, different short-chain fatty acid-producing bacteria may have divergent effects on Treg/Th17 balance, barrier stability, and synovial inflammatory responses, whereas current studies remain limited in their analysis of strain-specific and functional differences. Therefore, future investigations should move beyond merely evaluating symptomatic improvement and instead incorporate microbial functional characteristics, immune phenotypes, and metabolic profiles to enable more precise stratified assessment of different microbiota-targeted intervention strategies.

Future studies should further develop the “gut microbiota–immune–joint axis” framework by integrating longitudinal cohort analyses, mechanistic experiments, and interventional studies, while combining multi-omics approaches, immune phenotyping, metabolomics, and imaging indicators to dynamically evaluate the temporal relationships among microbial alterations, immune regulation, and structural joint changes. Overall, re-examining OA from the perspective of microbiota–immune interactions may not only deepen our understanding of disease pathogenesis, but also provide an important theoretical foundation for the development of novel precision therapeutic strategies based on microbiota modulation.

## References

[ref1] Abubakari A. Salvador-Oke K. T. M-Kamal H. S. Johnson A. M. (2025). Microbial membrane vesicles and the intestinal epithelium: a crosstalk in health and disease. Arch. Microbiol. 208:3. doi: 10.1007/s00203-025-04562-2, 41191120

[ref2] Acharya B. Tofthagen M. Maciej-Hulme M. L. Suissa M. R. Karlsson N. G. (2024). Limited support for a direct connection between prebiotics and intestinal permeability – a systematic review. Glycoconj. J. 41, 323–342. doi: 10.1007/s10719-024-10165-8, 39287885 PMC11522178

[ref3] Akhter S. Tasnim F. M. Islam M. N. Rauf A. Mitra S. Emran T. B. . (2023). Role of Th17 and IL-17 cytokines on inflammatory and auto-immune diseases. Curr. Pharm. Des. 29, 2078–2090. doi: 10.2174/1381612829666230904150808, 37670700

[ref9001] Álvarez Calatayud G. Leis Trabazo R. Seoane Reula E. Marcos Sánchez A. (2025). La microbiota intestinal y su modulación: impacto en la salud digestiva y en el sistema inmunitario [Gut microbiota and its modulation: impact on digestive health and immune system]. Nutr Hosp. 42, 51–55. doi: 10.20960/nh.06092 40728467

[ref4] Andrani M. Ferrari L. Borghetti P. Cavalli V. de Angelis E. Ravanetti F. . (2024). Short-chain fatty acids modulate the IPEC-J2 cell response to pathogenic *E. coli* LPS-activated PBMC. Res. Vet. Sci. 171:105231. doi: 10.1016/j.rvsc.2024.105231, 38513460

[ref5] Arias S. L. van Wijngaarden E. W. Balint D. Jones J. Crawford C. C. Shukla P. J. . (2025). Environmental factors drive bacterial degradation of gastrointestinal mucus. NPJ Biofilms Microbiomes 11:133. doi: 10.1038/s41522-025-00741-7 40670389 PMC12267731

[ref6] Arneth B. (2026). Immunometabolism of T cells and macrophages: human translational perspectives. Immunobiology 231:153151. doi: 10.1016/j.imbio.2025.153151, 41496197

[ref7] Ayushman M. Lee H. P. Agarwal P. Mikos G. Tong X. Jones S. . (2025). Sliding hydrogels reveal the modulation of Mechanosensing attenuates the inflammatory phenotype of osteoarthritic chondrocytes in 3D. J. Biomed. Mater. Res. A 113:e37861. doi: 10.1002/jbm.a.37861, 39718447 PMC12790461

[ref8] Bao-Anh N. Yang X. Hang H. (2025). Bile acid modulation of host immunity. Ann. N. Y. Acad. Sci. 1551, 63–83. doi: 10.1111/nyas.70014, 40778610

[ref9] Boem F. Lamminpää I. Amedei A. (2025). Updating the discontinuity theory to the extended immunity: the Symmunobiome concept. Eur. J. Immunol. 55:e202451528. doi: 10.1002/eji.202451528, 40251928 PMC12008767

[ref10] Cai H. Zhang J. Liu C. le T. N. Lu Y. Feng F. . (2024). High-fat diet-induced decreased circulating bile acids contribute to obesity associated with gut microbiota in mice. Foods 13:699. doi: 10.3390/foods13050699, 38472812 PMC10931208

[ref11] Carreto-Binaghi L. E. Sztein M. B. Booth J. S. (2024). Role of cellular effectors in the induction and maintenance of IgA responses leading to protective immunity against enteric bacterial pathogens. Front. Immunol. 15:1446072. doi: 10.3389/fimmu.2024.1446072, 39324143 PMC11422102

[ref12] Chasov V. Zmievskaya E. Ganeeva I. Gilyazova E. Davletshin D. Filimonova M. . (2024). Systemic lupus erythematosus therapeutic strategy: from immunotherapy to gut microbiota modulation. J. Biomed. Res. 38, 531–546. doi: 10.7555/JBR.38.20240009, 38828853 PMC11629155

[ref13] Chen B. Guan L. Wu C. Gong Y. Wu L. Zhang M. . (2025). Gut microbiota-butyrate-PPARγ Axis modulates adipose regulatory T cell population. Adv. Sci. (Weinh). 12:e2411086. doi: 10.1002/advs.202411086, 39998325 PMC12120792

[ref14] Chen R. Liu M. Jiang Q. Meng X. Wei J. (2023). The cyclic guanosine monophosphate synthase-stimulator of interferon genes pathway as a potential target for tumor immunotherapy. Front. Immunol. 14:1121603. doi: 10.3389/fimmu.2023.1121603 37153627 PMC10160662

[ref15] Chen J. Yang H. Qin Y. Zhou X. Ma Q. (2024). Tryptophan ameliorates metabolic syndrome by inhibiting intestinal Farnesoid X receptor signaling: the role of gut microbiota-bile acid crosstalk. Research (Wash D C) 7:0515. doi: 10.34133/research.0515 39679283 PMC11638488

[ref16] Dang Y. Liu Y. Zhang B. Zhang X. (2025). Aging microenvironment in osteoarthritis focusing on early-stage alterations and targeted therapies. Bone Res. 13:84. doi: 10.1038/s41413-025-00465-6 41073390 PMC12514018

[ref17] de Sire A. Mancuso E. Marotta N. Massimino M. Zito R. Averta C. . (2025). Association between gut microbiota composition and physical functioning in patients with knee osteoarthritis: a machine learning study. Sci. Rep. 15:40826. doi: 10.1038/s41598-025-24500-y 41258193 PMC12630774

[ref18] Del Sordo L. Blackler G. B. Philpott H. T. Riviere J. Gunaratnam L. Heit B. . (2023). Impaired Efferocytosis by synovial macrophages in patients with knee osteoarthritis. Arthritis Rheumatol. 75, 685–696. doi: 10.1002/art.42412, 36448607

[ref19] Deng C. Chen D. Yang L. Zhang Y. Jin C. Li Y. . (2025). The role of cGAS-STING pathway ubiquitination in innate immunity and multiple diseases. Front. Immunol. 16:1522200. doi: 10.3389/fimmu.2025.1522200, 40028324 PMC11868049

[ref20] Deng Y. Liu J. Bamahel A. S. Zhang P. Yu A. Cui H. . (2026). Chondrocyte inflammatory-immune responses in osteoarthritis: from pathogenesis to therapeutic intervention. Int. Immunopharmacol. 173:116270. doi: 10.1016/j.intimp.2026.116270, 41605051

[ref21] Di Cicco G. Marzano E. Mastrostefano A. Pitocco D. Castilho R. S. Zambelli R. . (2024). The Pathogenetic role of RANK/RANKL/OPG signaling in osteoarthritis and related targeted therapies. Biomedicine 12:2292. doi: 10.3390/biomedicines12102292 PMC11505501 39457605

[ref22] Ding G. Yang X. Li Y. Wang Y. du Y. Wang M. . (2025). Gut microbiota regulates gut homeostasis, mucosal immunity and influences immune-related diseases. Mol. Cell. Biochem. 480, 1969–1981. doi: 10.1007/s11010-024-05077-y, 39060829

[ref23] Dreo B. Muralikrishnan A. S. Husic R. Lackner A. Brügmann T. Haudum P. . (2024). JAK/STAT signaling in rheumatoid arthritis leukocytes is uncoupled from serum cytokines in a subset of patients. Clin. Immunol. 264:110238. doi: 10.1016/j.clim.2024.110238, 38729230

[ref24] Duan X. Boo Z. Z. Chua S. L. Chong K. H. C. Long Z. Yang R. . (2023). A bacterial quorum sensing regulated protease inhibits host immune responses by cleaving death domains of innate immune adaptors. Adv. Sci. (Weinh). 10:e2304891. doi: 10.1002/advs.202304891, 37870218 PMC10700182

[ref25] Edalat S. G. Gerber R. Houtman M. Lückgen J. Teixeira R. L. Palacios Cisneros M. D. P. . (2024). Molecular maps of synovial cells in inflammatory arthritis using an optimized synovial tissue dissociation protocol. iScience 27:109707. doi: 10.1016/j.isci.2024.109707, 38832018 PMC11144743

[ref26] Endo Y. Kanno T. Nakajima T. (2022). Fatty acid metabolism in T-cell function and differentiation. Int. Immunol. 34, 579–587. doi: 10.1093/intimm/dxac025 35700102

[ref27] Eriksen E. Graff P. Afanou A. K. (2026). Bacterial communities as modulators of innate immune Signalling: an *in vitro* perspective on toll-like receptor activation. Environ. Microbiol. Rep. 18:e70289. doi: 10.1111/1758-2229.70289, 41637678 PMC12872116

[ref28] Feng J. J. Maddirala N. R. Saint Fleur A. Zhou F. Yu D. Wei F. . (2025). Gut microbiome and immune system crosstalk in chronic inflammatory diseases: a narrative review of mechanisms and therapeutic opportunities. Microorganisms 13:2516. doi: 10.3390/microorganisms13112516 41304202 PMC12654787

[ref29] Fioretto L. Ziaco M. Nuzzo G. Albiani F. Saponaro M. Carbone D. . (2025). Small molecules as ligands in the tuning of immune regulatory receptors. Front. Immunol. 16:1648691. doi: 10.3389/fimmu.2025.1648691, 40948752 PMC12426008

[ref30] Fiorucci S. Marchianò S. Urbani G. di Giorgio C. Distrutti E. Zampella A. . (2024). Immunology of bile acids regulated receptors. Prog. Lipid Res. 95:101291. doi: 10.1016/j.plipres.2024.101291, 39122016

[ref31] Fortuna R. Wang W. Mayengbam S. Tuplin E. W. N. Sampsell K. Sharkey K. A. . (2024). Effect of prebiotic fiber on physical function and gut microbiota in adults, mostly women, with knee osteoarthritis and obesity: a randomized controlled trial.European. J. Nutr. 63, 2149–2161. doi: 10.1007/s00394-024-03415-w 38713231

[ref32] Gaspar M. G. Núñez-Carro C. Blanco-Blanco M. Blanco F. J. de Andrés M. C. (2025). Inflammaging contributes to osteoarthritis development and human microbiota variations and vice versa: a systematic review. Osteoarthr. Cartil. 33, 218–230. doi: 10.1016/j.joca.2024.11.005, 39612977

[ref33] Gates M. Schumacher S. M. Doyle W. J. Sofaly N. Roullet J. B. Ochoa-Repáraz J. (2026). Farnesol, the farnesol pathway, and the immune-gut-brain axis. Front. Pharmacol. 17:1718322. doi: 10.3389/fphar.2026.1718322, 41782929 PMC12953405

[ref34] Gilat R. Yazdi A. A. Weissman A. C. Joyce K. M. Bouftas F. A. Muth S. A. . (2025). The gut microbiome and joint microbiome show alterations in patients with knee osteoarthritis versus controls: a systematic review. Arthroscopy 41, 1226–1238. doi: 10.1016/j.arthro.2024.05.010, 38797504

[ref35] Gryaznova M. Burakova I. Smirnova Y. Morozova P. Chirkin E. Gureev A. . (2024). Effect of probiotic Bacteria on the gut microbiome of mice with lipopolysaccharide-induced inflammation. Microorganisms 12:1341. doi: 10.3390/microorganisms12071341, 39065109 PMC11278525

[ref36] Guo L. Ding Y. Li X. Liu X. Zang Z. Ji R. . (2026). Extraction, purification, structural characterization, and biological activity of polysaccharides from Dendrobium huoshanense: a review. Int. J. Biol. Macromol. 360:151918. doi: 10.1016/j.ijbiomac.2026.151918, 41962709

[ref37] Guo J. Lu M. Wang C. Wang D. Ma T. (2025). Nucleic acid diversity in cGAS-STING pathway activation and immune dysregulation. Biomedicine 13:2158. doi: 10.3390/biomedicines13092158 PMC12467401 41007720

[ref38] Guo S. Zhao J. Zhu R. Fan Z. Liu S. Wu W. (2025). Epigenetic modifications of gut microbiota and their potential role in atherosclerosis. Front. Pharmacol. 16:1638240. doi: 10.3389/fphar.2025.1638240, 40740992 PMC12307311

[ref39] Hamoudi C. Lashgari A. Aoudjit F. (2026). Integrins α3 and α6 promote Th17 cell migration by activating the purinergic receptor P2X4. Purinergic Signal 22:8. doi: 10.1007/s11302-025-10126-2 41557138 PMC12819906

[ref40] Hao X. Zhao J. Jia L. Ding G. Liang X. Su F. . (2025). LATS1-modulated ZBTB20 perturbing cartilage matrix homeostasis contributes to early-stage osteoarthritis. Bone Res. 13:33. doi: 10.1038/s41413-025-00414-3, 40069162 PMC11897192

[ref41] Haowen Y. Yuhan Y. Yuanyuan L. Xibin M. Yuxin W. Lingyun X. . (2025). Inhibitor of differentiation-2 protein ameliorates complete Freund's adjuvant-induced arthritis and inhibits STAT3 phosphorylation in the synovium. Immunol. Lett. 275:107008. doi: 10.1016/j.imlet.2025.107008, 40194667

[ref42] He Y. Shaoyong W. Chen Y. Li M. Gan Y. Sun L. . (2026). The functions of gut microbiota-mediated bile acid metabolism in intestinal immunity. J. Adv. Res. 80, 351–370. doi: 10.1016/j.jare.2025.05.015, 40354934 PMC12869224

[ref43] He X. Wedn A. Wang J. Gu Y. Liu H. Zhang J. . (2024). IUPHAR ECR review: the cGAS-STING pathway: novel functions beyond innate immune and emerging therapeutic opportunities. Pharmacol. Res. 201:107063. doi: 10.1016/j.phrs.2024.107063, 38216006

[ref44] Helm E. Y. Zhou L. (2023). Transcriptional regulation of innate lymphoid cells and T cells by aryl hydrocarbon receptor. Front. Immunol. 14:1056267. doi: 10.3389/fimmu.2023.1056267, 37056785 PMC10089284

[ref45] Holub M. N. Wahhab A. Rouse J. R. Danner R. McClune M. Dressler J. M. . (2023). Peptidoglycan in osteoarthritis synovial tissue is associated with joint inflammation. Preprint. Res Sq.:rs.3.rs-2842385. doi: 10.21203/rs.3.rs-2842385/v1, 38532447 PMC10967045

[ref46] Hou X. Chen Y. Chen F. Yin Y. Xu K. (2025). Taking intestinal microbes and the immune system as opportunities to maintain the intestinal health of piglets: review. Anim. Biosci. 38, 1827–1840. doi: 10.5713/ab.25.0100, 40400191 PMC12415457

[ref47] Hu K. Song M. Song T. Jia X. Song Y. (2025). Osteoimmunology in osteoarthritis: unraveling the interplay of immunity, inflammation, and joint degeneration. J. Inflamm. Res. 18, 4121–4142. doi: 10.2147/JIR.S514002, 40125089 PMC11930281

[ref48] Huang H. Yang C. Li S. Zhan H. Tan J. Chen C. . (2025). Lizhong decoction alleviates experimental ulcerative colitis via regulating gut microbiota-SCFAs-Th17/Treg axis. J. Ethnopharmacol. 349:119958. doi: 10.1016/j.jep.2025.119958, 40350047

[ref49] Hutton J. Sun W. Hasegawa T. (2025). The ontogeny of synovial tissue macrophages. Front. Immunol. 16:1603473. doi: 10.3389/fimmu.2025.1603473 40463391 PMC12129757

[ref50] Jan P. Nikola M. Liliana T. Karolina K. Valéria G. Zdeněk Z. . (2025). Microbiota modulate immune cell populations and drive dynamic structural changes in gut-associated lymphoid tissue. Gut Microbes 17:2543908. doi: 10.1080/19490976.2025.2543908, 40802565 PMC12351735

[ref51] Jevtić B. Stegnjaić G. Stanisavljević S. Lazarević M. Nikolić F. Fraser G. L. . (2025). Amelioration of central nervous system autoimmunity through FFAR2 Agonism is associated with changes in gut microbiota. Brain Behav. 15:e70350. doi: 10.1002/brb3.70350, 40021945 PMC11870826

[ref52] Jing Y. Wang K. Pi T. Chen Z. Liu T. Liu X. . (2025). Crucial role of low molecular weight chondroitin sulfate from hybrid sturgeon cartilage in osteoarthritis improvement: focusing on apoptosis, systemic inflammation, and intestinal flora. Int. J. Biol. Macromol. 298:139850. doi: 10.1016/j.ijbiomac.2025.139850, 39814287

[ref53] Kang J. Zhang L. Zhang L. Nan N. Liu Y. Hao H. (2025). Exosomal miR-574-3p from adipose-derived mesenchymal stem modulates CRIM1/BMPs signaling to restrain chondrocytes hypertrophy and inflammatory response in knee osteoarthritis. Int. Immunopharmacol. 159:114916. doi: 10.1016/j.intimp.2025.114916, 40435818

[ref54] Kanno T. Nakajima T. Miyako K. Endo Y. (2023). Lipid metabolism in Th17 cell function. Pharmacol. Ther. 245:108411. doi: 10.1016/j.pharmthera.2023.108411, 37037407

[ref55] Keshavarz M. Franz M. Xie H. Zanchi C. Mbedi S. Sparmann S. . (2025). Immune-mediated indirect interaction between gut microbiota and bacterial pathogens. BMC Biol. 23:278. doi: 10.1186/s12915-025-02399-1 40958093 PMC12442303

[ref56] Kim C. H. (2023). Complex regulatory effects of gut microbial short-chain fatty acids on immune tolerance and autoimmunity. Cell. Mol. Immunol. 20, 341–350. doi: 10.1038/s41423-023-00987-1, 36854801 PMC10066346

[ref57] Kouraki A. Franks S. Vijay A. Kurien T. Taylor M. A. Smith S. L. . (2026). Effect of prebiotic supplementation with and without physiotherapy on pain and pain sensitivity in people with knee osteoarthritis. Nutrients 18:714. doi: 10.3390/nu18050714, 41829888 PMC12986947

[ref58] Kumari B. Kumari U. Singh D. K. Husain G. M. Patel D. K. Shakya A. . (2024). Molecular targets of Valeric acid: a bioactive natural product for endocrine, metabolic, and immunological disorders. Endocr. Metab. Immune Disord. Drug Targets 24, 1506–1517. doi: 10.2174/0118715303262653231120043819, 38375842

[ref59] Lata K. Gandhi S. Chatterjee S. Puravankara S. Chauhan A. Nandy K. . (2026). Bacterial pore-forming toxins: mechanisms and implications for host immunity. Biosci. Rep. 46:BSR20250103. doi: 10.1042/BSR20250103, 41866932 PMC13238964

[ref60] Li H. Breedijk A. Dietrich N. Jarczyk J. Nuhn P. Krämer B. K. . (2023). Lipopolysaccharide tolerance in human primary monocytes and polarized macrophages. Int. J. Mol. Sci. 24:12196. doi: 10.3390/ijms241512196 37569572 PMC10419197

[ref61] Li N. Guo X. (2026). The gut microbiota and host immunity synergistically orchestrate colonization resistance. Gut Microbes 18:2611545. doi: 10.1080/19490976.2025.2611545, 41482864 PMC12773493

[ref62] Li T. Li Q. Liu S. Cao J. Mei J. Gong J. . (2025). Targeted V-type peptide-decorated nanoparticles prevent colitis by inhibiting endosomal TLR signaling and modulating intestinal macrophage polarization. Biomaterials 314:122843. doi: 10.1016/j.biomaterials.2024.122843, 39321686

[ref63] Liang Y. Wang Y. Xing J. Li J. Zhang K. (2025). The regulatory mechanisms and treatment of HDAC6 in immune dysregulation diseases. Front. Immunol. 16:1653588. doi: 10.3389/fimmu.2025.1653588, 41058704 PMC12497803

[ref64] Lin Z. Tang H. Che L. Chen M. Ye Z. Yang X. . (2026). Structural analysis and immunomodulatory properties of lipopolysaccharide from the gut symbiont Phocaeicola dorei. Carbohydr. Polym. 373:124566. doi: 10.1016/j.carbpol.2025.124566, 41320355

[ref65] Lisicka W. Earley Z. M. Sifakis J. J. Erickson S. A. Mattingly J. R. Wu-Woods N. J. . (2025). Immunoglobulin A controls intestinal virus colonization to preserve immune homeostasis. Cell Host Microbe 33, 498–511.e10. doi: 10.1016/j.chom.2025.03.004, 40154490 PMC12235565

[ref66] Liu H. Sun X. Li Y. Tang Z. Hou Y. Xia Q. . (2025). Peptidoglycan recognition protein L1 regulates intestinal immunity and microbial homeostasis via the IMD pathway in the silkworm, *Bombyx mori*. Insect Sci. doi: 10.1111/1744-7917.70178, 41147042

[ref67] Liu C. Yi X. Wang P. Wang P. Li Y. Xu H. . (2025). Tinosporine alleviates collagen-induced arthritis in rats via modulating the gut microbiota-metabolome-immunity axis. Int. Immunopharmacol. 157:114752. doi: 10.1016/j.intimp.2025.114752, 40300353

[ref68] Lönsjö J. Rydén M. Turkiewicz A. Hughes V. Tjörnstand J. Önnerfjord P. . (2025). Altered co-expression patterns of synovial fluid proteins related to the immune system and extracellular matrix organization in late stage OA, compared to non-OA controls. Front. Immunol. 16:1523103. doi: 10.3389/fimmu.2025.1523103, 40625747 PMC12229868

[ref69] Lu J. Yu M. Li J. (2024). PKC-δ promotes IL-1β-induced apoptosis of rat chondrocytes and via activating JNK and P38 MAPK pathways. Cartilage 15, 315–327. doi: 10.1177/19476035231181446, 37491820 PMC11418514

[ref71] Luo M. Li S. Yang Y. Sun J. Su Y. Huang D. . (2024). Effects of Salmonella outer membrane vesicles on intestinal microbiota and intestinal barrier function. Foodborne Pathog. Dis. 21, 257–267. doi: 10.1089/fpd.2023.0096, 38215267

[ref72] Lv J. Hao P. Zhou Y. Liu T. Wang L. Song C. . (2025). Role of the intestinal flora-immunity axis in the pathogenesis of rheumatoid arthritis-mechanisms regulating short-chain fatty acids and Th17/Treg homeostasis. Mol. Biol. Rep. 52:617. doi: 10.1007/s11033-025-10714-w, 40544212

[ref73] Ma Z. F. Lee Y. Y. (2025). The role of the gut microbiota in health, diet, and disease with a focus on obesity. Foods 14:492. doi: 10.3390/foods14030492, 39942085 PMC11817362

[ref74] Ma J. Liu X. Zhao Y. Lu Q. Ding G. Wang Y. . (2025). Th17/Treg balance is regulated during the suppression of experimental autoimmune encephalomyelitis treated by Astragalus polysaccharides via the microbiota-gut-brain axis. Brain Res. Bull. 220:111171. doi: 10.1016/j.brainresbull.2024.111171, 39675488

[ref75] Maciel-Fiuza M. F. Muller G. C. Campos D. M. S. do Socorro Silva Costa P. Peruzzo J. Bonamigo R. R. . (2023). Role of gut microbiota in infectious and inflammatory diseases. Front. Microbiol. 14:1098386. doi: 10.3389/fmicb.2023.1098386, 37051522 PMC10083300

[ref76] Mansoori Moghadam Z. Zhao B. Raynaud C. Strohmeier V. Neuber J. Lösslein A. K. . (2025). Reactive oxygen species regulate early development of the intestinal macrophage-microbiome interface. Blood 145, 2025–2040. doi: 10.1182/blood.2024025240, 39899882

[ref77] Mårtensson J. Björkman L. Lind S. Viklund M. B. Zhang L. Gutierrez S. . (2023). The ketone body acetoacetate activates human neutrophils through FFAR2. J. Leukoc. Biol. 113, 577–587. doi: 10.1093/jleuko/qiad035, 36999365

[ref78] Meléndez-Oliva E. Martínez-Pozas O. Sinatti P. Carreras-Presas C. M. Cuenca-Zaldívar J. N. Turroni S. . (2025). Relationship between the gut microbiome, tryptophan-derived metabolites, and osteoarthritis-related pain: a systematic review with meta-analysis. Nutrients 17:264. doi: 10.3390/nu17020264 39861394 PMC11767305

[ref79] Mobasheri A. Castro-Domínguez F. Gualillo O. Kluzek S. Ruiz S. P. Largo R. (2026). Targeting synovial inflammation in knee osteoarthritis: translational insights for diagnosis and therapy. J. Orthop. Translat. 56:101012. doi: 10.1016/j.jot.2025.10.004, 41836546 PMC12988513

[ref80] Mohanty I. Allaband C. Mannochio-Russo H. el Abiead Y. Hagey L. R. Knight R. . (2024). The changing metabolic landscape of bile acids - keys to metabolism and immune regulation. Nat. Rev. Gastroenterol. Hepatol. 21, 493–516. doi: 10.1038/s41575-024-00914-3, 38575682 PMC12248421

[ref81] Mohr A. E. Crawford M. Jasbi P. Fessler S. Sweazea K. L. (2022). Lipopolysaccharide and the gut microbiota: considering structural variation. FEBS Lett. 596, 849–875. doi: 10.1002/1873-3468.14328, 35262962

[ref82] Moulin D. Sellam J. Berenbaum F. Guicheux J. Boutet M. A. (2025). The role of the immune system in osteoarthritis: mechanisms, challenges and future directions. Nat. Rev. Rheumatol. 21, 221–236. doi: 10.1038/s41584-025-01223-y, 40082724

[ref83] Narla A. V. Hwa T. Murugan A. (2025). Dynamic coexistence driven by physiological transitions in microbial communities. Proc. Natl. Acad. Sci. USA 122:e2405527122. doi: 10.1073/pnas.240552712, 40244660 PMC12037064

[ref84] Nie Y. Lin T. Yang Y. Liu W. Hu Q. Chen G. . (2024). The downregulation of tight junction proteins and pIgR in the colonic epithelium causes the susceptibility of EpCAM+/− mice to colitis and gut microbiota dysbiosis. Front. Mol. Biosci. 11:1442611. doi: 10.3389/fmolb.2024.1442611, 39188786 PMC11345229

[ref85] Ning P. Lin S. Shi Y. Liu T. (2025). Potential role of gut-related factors in the pathology of cartilage in osteoarthritis. Front. Nutr. 11:1515806. doi: 10.3389/fnut.2024.1515806, 39845920 PMC11753001

[ref86] Niu L. Chen W. Yin Z. Tan H. Cui J. Su J. (2025). Bacterial extracellular vesicles in osteoarthritis: a new bridge of the gut-joint axis. Gut Microbes 17:2489069. doi: 10.1080/19490976.2025.2489069, 40213946 PMC12716045

[ref87] Okumura R. Takeda K. (2024). The role of the mucosal barrier system in maintaining gut symbiosis to prevent intestinal inflammation. Semin. Immunopathol. 47:2. doi: 10.1007/s00281-024-01026-5 39589551 PMC11599372

[ref88] Oleszycka E. O'Brien E. C. Freeley M. Lavelle E. C. Long A. (2023). Bile acids induce IL-1α and drive NLRP3 inflammasome-independent production of IL-1β in murine dendritic cells. Front. Immunol. 14:1285357. doi: 10.3389/fimmu.2023.1285357, 38090554 PMC10711081

[ref89] Pang S. Wang L. Liu M. Shao M. Zhu G. Duan Q. (2025). Truncated flagellin lacking the hypervariable region: a structural basis for improved immune responses and adjuvanticity. Int. J. Biol. Macromol. 308:142742. doi: 10.1016/j.ijbiomac.2025.142742, 40180103

[ref90] Panichi V. Costantini S. Grasso M. Arciola C. R. Dolzani P. (2024). Innate immunity and synovitis: key players in osteoarthritis progression. Int. J. Mol. Sci. 25:12082. doi: 10.3390/ijms252212082, 39596150 PMC11594236

[ref91] Parada-Venegas D. de M. Dubois-Camacho K. Landskron G. Blokzijl T. Molina H. . (2025). Butyrate suppresses mucosal inflammation in inflammatory bowel disease primarily through HDAC3 inhibition in monocytes and macrophages. FEBS J. 292, 6134–6157. doi: 10.1111/febs.70289, 41110099 PMC12631161

[ref92] Pereira A. P. R. Jacobowski A. C. Almeida C. V. de Araújo Boleti A. P. Pereira R. A. Matos C. O. . (2026). A computationally optimized peptide (KI17) derived from Talisia esculenta with potent action against multidrug-resistant pathogens. Biochim. Biophys. Acta Gen. Subj. 1870:130946. doi: 10.1016/j.bbagen.2026.130946, 41935703

[ref93] Perrin L. Gannavarapu V. R. Pérez-González C. Rivera C. Descroix S. Lennon-Duménil A. M. . (2025). The Arp2/3 complex maintains gut epithelial integrity under mechanical challenge. Curr. Biol. 35, 4827–4836.e5. doi: 10.1016/j.cub.2025.08.026, 40930096

[ref94] Pham T. H. Monack D. M. (2023). Turning foes into permissive hosts: manipulation of macrophage polarization by intracellular bacteria. Curr. Opin. Immunol. 84:102367. doi: 10.1016/j.coi.2023.102367, 37437470 PMC10543482

[ref95] Pilapitiya A. U. Hor L. Pan J. Wijeyewickrema L. C. Pike R. N. Leyton D. L. . (2025). The crystal structure of the toxin EspC from enteropathogenic *Escherichia coli* reveals the mechanism that governs host cell entry and cytotoxicity. Gut Microbes 17:2483777. doi: 10.1080/19490976.2025.2483777, 40164999 PMC11970781

[ref96] Piva F. Gervois P. Karrout Y. Sané F. Romond M. B. (2023). Gut-joint Axis: impact of Bifidobacterial Cell Wall lipoproteins on arthritis development. Nutrients 15:4861. doi: 10.3390/nu15234861 38068720 PMC10708502

[ref97] Plewa P. Graczyk P. Figiel K. Dach A. Pawlik A. (2026). Gut and joint microbiome and Dysbiosis: a new perspective on the pathogenesis and treatment of osteoarthritis. Pathogens 15:62. doi: 10.3390/pathogens15010062, 41599046 PMC12845289

[ref98] Pradeep A. Mathew A. I. Vemula P. K. Bhat S. G. Narayanan S. (2024). Investigating the pro-inflammatory differentiation of macrophages with bacterial ghosts in potential infection control. Arch. Microbiol. 206:361. doi: 10.1007/s00203-024-04089-y 39066807 PMC7616332

[ref99] Pueyo MolinerA. ItoK. ZauckeF. KellyD. J. de RuijterM. MaldaJ. (2025). Restoring articular cartilage: insights from structure, composition and development. Nat. Rev. Rheumatol. 21, 291–308. doi: 10.1038/s41584-025-01236-7, 40155694

[ref100] Qiu H. Wen Y. Luo Y. Lv S. Huang J. Chen B. . (2025). Gut microbiota regulates serum metabolites in mice with nonalcoholic fatty liver disease via gut metabolites: mechanisms involving branched-chain amino acids and unsaturated fatty acids. Front. Endocrinol. (Lausanne) 16:1606669. doi: 10.3389/fendo.2025.1606669 40766290 PMC12321561

[ref101] Ren J. Jiang X. Yu G. Wu W. Chen M. Zhao Y. . (2026). Sulforaphane ameliorates DSS-induced colitis and secondary liver injury in mice: proposed mechanism in the SCFAs-FFAR2/3-macrophage polarization axis. J. Nutr. Biochem. 150:110239. doi: 10.1016/j.jnutbio.2025.110239, 41412441

[ref102] Richie T. Lee S. T. M. (2025). Decoding the gut microbiota: mechanisms of host-microbe interactions and inflammatory pathologies. Digestion 17, 1–18. doi: 10.1159/000549457 41248092

[ref103] Saadh M. J. Allela O. Q. B. Ballal S. Mahdi M. S. Chahar M. Verma R. . (2025). The effects of microbiota-derived short-chain fatty acids on T lymphocytes: from autoimmune diseases to cancer. Semin. Oncol. 52:152398. doi: 10.1016/j.seminoncol.2025.152398, 40834607

[ref104] Sadeghi Shermeh A. Royzman D. Kuhnt C. Draßner C. Stich L. Steinkasserer A. . (2023). Differential modulation of dendritic cell biology by endogenous and exogenous aryl hydrocarbon receptor ligands. Int. J. Mol. Sci. 24:7801. doi: 10.3390/ijms24097801, 37175508 PMC10177790

[ref105] Saha K. Zhou Y. Turner J. R. (2025). Tight junction regulation, intestinal permeability, and mucosal immunity in gastrointestinal health and disease. Curr. Opin. Gastroenterol. 41, 46–53. doi: 10.1097/MOG.0000000000001066, 39560621 PMC11620928

[ref106] Sawoo R. Bishayi B. (2024). TLR4/TNFR1 blockade suppresses STAT1/STAT3 expression and increases SOCS3 expression in modulation of LPS-induced macrophage responses. Immunobiology 229:152840. doi: 10.1016/j.imbio.2024.152840, 39126792

[ref107] Schonfeldova B. Chibwana M. Gentek R. Zec K. Udalova I. A. (2025). Perivascular RELMα-positive synovial macrophages recruit monocytes at the onset of inflammatory arthritis. Front. Immunol. 16:1567661. doi: 10.3389/fimmu.2025.1567661, 40313955 PMC12043459

[ref108] Segui-Perez C. Stapels D. A. C. Ma Z. Su J. Passchier E. Westendorp B. . (2024). MUC13 negatively regulates tight junction proteins and intestinal epithelial barrier integrity via protein kinase C. J. Cell Sci. 137:jcs261468. doi: 10.1242/jcs.261468, 38345099 PMC10984281

[ref109] Selvakumar B. Samsudin R. (2025). Intestinal barrier dysfunction in inflammatory bowel disease: pathophysiology to precision therapeutics. Inflamm. Bowel Dis. 31, 3450–3464. doi: 10.1093/ibd/izaf225, 41189113

[ref110] Seo B. Lim M. Y. (2025). Balancing harm and harmony: evolutionary dynamics between gut microbiota-derived flagellin and TLR5-mediated host immunity and metabolism. Virulence 16:2512035. doi: 10.1080/21505594.2025.2512035, 40444793 PMC12128667

[ref111] Sha Y. Zhang B. Chen L. Wang C. Sun T. (2022). Dehydrocorydaline accelerates cell proliferation and extracellular matrix synthesis of TNFα-treated human chondrocytes by targeting Cox2 through JAK1-STAT3 signaling pathway. Int. J. Mol. Sci. 23:7268. doi: 10.3390/ijms23137268, 35806272 PMC9267121

[ref112] Shaheen N. Khursheed W. Gurung B. Wang S. (2025). *Akkermansia muciniphila*: a key player in gut microbiota-based disease modulation. Microbiol. Res. 301:128317. doi: 10.1016/j.micres.2025.128317 40845731

[ref113] Shao T. Hsu R. Rafizadeh D. L. Wang L. Bowlus C. L. Kumar N. . (2023). The gut ecosystem and immune tolerance. J. Autoimmun. 141:103114. doi: 10.1016/j.jaut.2023.103114, 37748979

[ref114] Shao-Yu Y. Niu D. Chen J. Li W. Y. Wang X. Meng Q. W. . (2025). Antibiotic cocktail-induced changes in gut microbiota drive alteration of bile acid metabolism to restrain Th17 differentiation through the FXR-NLRP3 axis. Gut Microbes 17:2582944. doi: 10.1080/19490976.2025.2582944, 41305918 PMC12667630

[ref115] Sharma A. Sharma G. Im S. H. (2025). Gut microbiota in regulatory T cell generation and function: mechanisms and health implications. Gut Microbes 17:2516702. doi: 10.1080/19490976.2025.2516702, 40517372 PMC12169050

[ref116] Shen Q. Huang W. Qiu Y. Wang S. Zhang B. Sun N. . (2023). Bergapten exerts a chondroprotective effect in temporomandibular joint osteoarthritis by combining intestinal flora alteration and reactive oxygen species reduction. Biomed. Pharmacother. 167:115525. doi: 10.1016/j.biopha.2023.115525, 37748407

[ref117] Sobacchi C. Menale C. Crisafulli L. Ficara F. (2025). Role of RANKL signaling in bone homeostasis. Physiology (Bethesda) 40. doi: 10.1152/physiol.00031.2024, 39255276

[ref118] Solvay M. Holfelder P. Klaessens S. Pilotte L. Stroobant V. Lamy J. . (2023). Tryptophan depletion sensitizes the AHR pathway by increasing AHR expression and GCN2/LAT1-mediated kynurenine uptake, and potentiates induction of regulatory T lymphocytes. J. Immunother. Cancer 11:e006728. doi: 10.1136/jitc-2023-006728, 37344101 PMC10314700

[ref119] Song G. Xie Y. Yi L. Cheng W. Jia H. Shi W. . (2025). Bile acids affect intestinal barrier function through FXR and TGR5. Front. Med. (Lausanne) 12:1607899. doi: 10.3389/fmed.2025.1607899 40692955 PMC12277261

[ref120] Stierschneider A. Wiesner C. (2023). Shedding light on the molecular and regulatory mechanisms of TLR4 signaling in endothelial cells under physiological and inflamed conditions. Front. Immunol. 14:1264889. doi: 10.3389/fimmu.2023.1264889, 38077393 PMC10704247

[ref121] Su J. Sun X. Chen X. Wei K. Luo D. Yang S. . (2025). CIP2A promotes inflammation and exacerbates osteoarthritis by targeting CEMIP. Cell. Mol. Biol. Lett. 30:67. doi: 10.1186/s11658-025-00748-0, 40490703 PMC12150572

[ref122] Sun N. Zhao Y. Zhang A. He Y. (2025). Gut microbiota and osteoarthritis: epidemiology, mechanistic analysis, and new horizons for pharmacological interventions. Front. Cell. Infect. Microbiol. 15:1605860. doi: 10.3389/fcimb.2025.1605860, 40740348 PMC12307352

[ref123] Svensson D. Nilsson B. O. (2025). Human antimicrobial/host defense peptide LL-37 may prevent the spread of a local infection through multiple mechanisms: an update. Inflamm. Res. 74:36. doi: 10.1007/s00011-025-02005-8, 40063262 PMC11893641

[ref124] Tian M. Hao F. Jin X. Wang X. Chang T. He S. . (2025). KLHL25-ACLY module functions as a switch in the fate determination of the differentiation of iTreg/Th17. Commun. Biol. 8:471. doi: 10.1038/s42003-025-07917-z, 40119138 PMC11928475

[ref125] Tian M. Zhu Y. Lu S. Qin Y. Li X. Wang T. . (2025). Clinical efficacy of probiotic supplementation in the treatment of knee osteoarthritis: a meta-analysis. Front. Microbiol. 16:1526690. doi: 10.3389/fmicb.2025.1526690, 40276226 PMC12020436

[ref126] Tran M. Huh J. R. Devlin A. S. (2025). The role of gut microbial metabolites in the T cell lifecycle. Nat. Immunol. 26, 1246–1257. doi: 10.1038/s41590-025-02227-2, 40691327 PMC13124069

[ref127] Tung N. T. C. Nogami M. Iwasaki M. Yahara Y. Seki S. Makino H. . (2025). M2-like macrophages derived from THP-1 cells promote myofibroblast differentiation of synovial fibroblasts in association with the TGF-β1/SMAD2/3 signaling pathway. Sci. Rep. 15:25505. doi: 10.1038/s41598-025-10858-6 40665033 PMC12264265

[ref128] Vonk L. A. (2025). Key insights and implications of cartilage degradation in osteoarthritis. Connect. Tissue Res. 66, 393–398. doi: 10.1080/03008207.2025.2536153, 40686393

[ref129] Wang L. (2025). Complement 3 and 4 impact in osteoarthritis. Biomark. Med 19, 81–90. doi: 10.1080/17520363.2024.2409062, 39893562 PMC11792862

[ref130] Wang S. Fan F. Wang S. Liu M. Wu J. Hu X. . (2026). Artemisia-derived carbon dots with excellent pharmacological activities and ROS scavenging for osteoarthritis treatment. J. Nanobiotechnol. 24:93. doi: 10.1186/s12951-026-04046-5 PMC12853929 41559762

[ref131] Wang J. Guo P. Wu D. Yi J. Jiang Q. Hu J. . (2025). Rejuvenating hyaline cartilage with senescence-targeting Si-ADAM19 delivery for osteoarthritis therapy. Adv. Sci. (Weinh). 12:e2414419. doi: 10.1002/advs.202414419, 39927476 PMC11967805

[ref132] Wang L. Y. He L. H. Xu L. J. Li S. B. (2024). Short-chain fatty acids: bridges between diet, gut microbiota, and health. J. Gastroenterol. Hepatol. 39, 1728–1736. doi: 10.1111/jgh.16619, 38780349

[ref133] Wang J. He M. Yang M. Ai X. (2024). Gut microbiota as a key regulator of intestinal mucosal immunity. Life Sci. 345:122612. doi: 10.1016/j.lfs.2024.122612, 38588949

[ref134] Wang W. Liu X. Nan H. Li H. Yan L. (2025). Specific gut microbiota and serum metabolite changes in patients with osteoarthritis. Front cell. Dev. Biol. 13:1543510. doi: 10.3389/fcell.2025.1543510, 40027098 PMC11868077

[ref135] Wang X. Liu Y. Sun Z. Li J. Lu Z. Huang J. . (2026). Multi-omics reveal the dysregulated gut-joint Axis in knee synovitis: data from two osteoarthritis studies in China. Adv. Sci. (Weinh). 13:e12020. doi: 10.1002/advs.202512020, 41354462 PMC12866873

[ref136] Wang W. Liu H. Zhang M. Wang L. (2026). Integrative multi-omics analysis reveals gut microbiota-derived metabolites and immune regulatory pathways in osteoarthritis pathogenesis. J. Orthop. Surg. Res. 21:87. doi: 10.1186/s13018-025-06604-3 41491956 PMC12870727

[ref137] Wang P. Qian H. Xiao M. Lv J. (2023). Role of signal transduction pathways in IL-1β-induced apoptosis: pathological and therapeutic aspects. Immun. Inflamm. Dis. 11:e762. doi: 10.1002/iid3.762, 36705417 PMC9837938

[ref138] Wang H. Sun J. Ma G. You F. Heng B. C. Bai Y. . (2025). The role and mechanism of bacterial outer membrane vesicles in the development of periodontitis. Front. Microbiol. 16:1654137. doi: 10.3389/fmicb.2025.1654137, 40969433 PMC12442421

[ref139] Wang W. Tao Y. Zhu M. (2026). Effects of functional dietary fiber supplementation combined with home-based exercise on gut microbiota diversity and low-grade inflammation in urban sedentary adults. Front. Nutr. 13:1769785. doi: 10.3389/fnut.2026.1769785, 41859657 PMC12997537

[ref140] Wang X. Xiao G. (2023). Recent advances in chemical synthesis of structural domains of lipopolysaccharides from the commensal gut-associated microbiota. Chembiochem 24:e202300552. doi: 10.1002/cbic.202300552, 37731010

[ref141] Wang Y. Yang R. Luo J. Li J. Huang B. Yang J. (2025). Bacterial outer membrane vesicles in immune modulation: from mechanisms to applications. J. Mater. Chem. B 13, 13867–13880. doi: 10.1039/d5tb01708d, 41058591

[ref142] Weaver D. F. (2024). Endogenous antimicrobial-immunomodulatory molecules: networking biomolecules of innate immunity. Chembiochem 25:e202400089. doi: 10.1002/cbic.202400089, 38658319

[ref143] Wen P. Liu L. (2025). Functions of macrophages, T cells, and neutrophils in the synovial microenvironment of osteoarthritis. J. Inflamm. Res. 18, 15671–15686. doi: 10.2147/JIR.S563253, 41243987 PMC12619582

[ref144] Wu D. Huang Y. Zhao J. Long W. Wang B. Wang Y. . (2025). Synovial macrophages drive severe joint destruction in established rheumatoid arthritis. Sci. Rep. 15:12111. doi: 10.1038/s41598-025-93784-x, 40204828 PMC11982296

[ref145] Wu X. Sun Q. Li X. Jiang L. Chen L. (2024). Halofuginone inhibits Osteoclastogenesis and enhances Osteoblastogenesis by regulating Th17/Treg cell balance in multiple myeloma mice with bone lesions. Indian J. Hematol. Blood Transfus. 40, 407–414. doi: 10.1007/s12288-024-01756-4, 39011260 PMC11246324

[ref146] Xi Y. Wang Z. Wei Y. Xiao N. Duan L. Zhao T. . (2025). Gut microbiota and osteoarthritis: from pathogenesis to novel therapeutic opportunities. Am. J. Chin. Med. 53, 43–66. doi: 10.1142/S0192415X2550003X 39880660

[ref147] Xia J. Shao Y. Li B. Wu T. He Z. Feng Z. . (2025). Integrative analysis of the gut microbiota, bile acid pathways, and immune dysregulation in dyslipidemia models. iScience 28:114001. doi: 10.1016/j.isci.2025.114001 41377665 PMC12686710

[ref148] Xie L. Gao F. Xu J. Xiong W. Yin J. Sun W. (2026). TREM1 enhances macrophage Proinflammatory response to LPS by promoting NF-κB activation via an IL-26-mediated JAK/STAT signaling pathway. Iran. J. Allergy Asthma Immunol. 25, 58–68. doi: 10.18502/ijaai.v25i1.20439, 41674173

[ref149] Yang X. Feng K. Wang S. Yuan M. M. Peng X. He Q. . (2024). Unveiling the deterministic dynamics of microbial meta-metabolism: a multi-omics investigation of anaerobic biodegradation. Microbiome 12:166. doi: 10.1186/s40168-024-01890-1 39244624 PMC11380791

[ref150] Yang Y. Hao C. Jiao T. Yang Z. Li H. Zhang Y. . (2025). Osteoarthritis treatment via the GLP-1-mediated gut-joint axis targets intestinal FXR signaling. Science 388:eadt0548. doi: 10.1126/science.adt0548, 40179178

[ref151] Yang G. M. Jiang H. Y. Ren S. H. Xu Y. N. Wang H. D. Shao B. . (2026). Oral microcapsules encapsulating endometrial regenerative cell-derived exosomes promote intestinal epithelial barrier repair and ameliorate experimental colitis. Int. J. Nanomedicine 21:540230. doi: 10.2147/IJN.S540230, 41907368 PMC13022928

[ref152] Yang S. Liu H. Liu Y. (2025). Advances in intestinal epithelium and gut microbiota interaction. Front. Microbiol. 16:1499202. doi: 10.3389/fmicb.2025.1499202, 40104591 PMC11914147

[ref153] Yang H. Shi X. Wang B. Li H. Li B. Zhou T. . (2025). Bile acid receptors regulate the role of intestinal macrophages in inflammatory bowel disease. Front. Immunol. 16:1577000. doi: 10.3389/fimmu.2025.1577000, 40599770 PMC12208833

[ref154] Yu F. Zhu C. Wu W. (2025). Senile osteoarthritis regulated by the gut microbiota: from mechanisms to treatments. Int. J. Mol. Sci. 26:1505. doi: 10.3390/ijms26041505, 40003971 PMC11855920

[ref155] Yuan Y. Liang X. Kang S. Liu Y. (2025). The mediation of circulating inflammatory proteins in the causal pathway from immune cells to osteoarthritis. J. Orthop. Surg. Res. 20:939. doi: 10.1186/s13018-025-06374-y, 41163231 PMC12574021

[ref156] Zammarchi I. Santacroce G. Puga-Tejada M. Hayes B. Crotty R. O’Driscoll E. . (2024). Epithelial neutrophil localization and tight junction Claudin-2 expression are innovative outcome predictors in inflammatory bowel disease. United European Gastroenterol J 12, 1155–1166. doi: 10.1002/ueg2.12677, 39361538 PMC11578851

[ref157] Zapata-Linares N. Toillon I. Wanherdrick K. Pigenet A. Duhalde F. Binvignat M. . (2025). Implication of bone marrow adipose tissue in bone homeostasis during osteoarthritis. Osteoarthr. Cartil. 33, 951–964. doi: 10.1016/j.joca.2025.03.004, 40154729

[ref158] Zec K. Schonfeldova B. Ai Z. van Grinsven E. Pirgova G. Eames H. L. . (2023). Macrophages in the synovial lining niche initiate neutrophil recruitment and articular inflammation. J. Exp. Med. 220:e20220595. doi: 10.1084/jem.20220595, 37115585 PMC10148166

[ref159] Zeng S. Wang S. Mu D. (2025). Metagenomics for IgA-coated gut microbiota: from taxonomy to function. Trends Microbiol. 33, 823–825. doi: 10.1016/j.tim.2025.04.001, 40246602

[ref160] Zhang S. Han Y. Schofield W. Nicosia M. Karell P. E. Newhall K. P. . (2023). Select symbionts drive high IgA levels in the mouse intestine. Cell Host Microbe 31, 1620–1638.e7. doi: 10.1016/j.chom.2023.09.001, 37776865

[ref161] Zhang Q. He X. Chen W. Jiu J. Gao C. Gao T. (2024). Vitamin D3 attenuates autoimmune thyroiditis by regulating Th17/Treg cell differentiation via YAP/JAK1/STAT1 axis. Immunol. Lett. 269:106890. doi: 10.1016/j.imlet.2024.106890, 38959983

[ref162] Zhang Y. He X. Yin D. Zhang Y. (2024). Redefinition of synovial fibroblasts in rheumatoid arthritis. Aging Dis. 16, 2054–2072. doi: 10.14336/AD.2024.0514, 39122458 PMC12221409

[ref163] Zhang A. Niu L. Ni Y. Liu W. Gao X. Chang L. . (2025). STAT3 inhibition mitigates experimental autoimmune gastritis by restoring Th17/Treg immune balance. Immunol. Res. 73:90. doi: 10.1007/s12026-025-09643-4, 40471463

[ref164] Zhang Y. Tian S. Wang R. Ning Y. (2026). Research progress on regulatory mechanisms of mucosal barriers and their applications in allergic diseases. Front. Immunol. 17:1671677. doi: 10.3389/fimmu.2026.1671677, 41676143 PMC12886358

[ref165] Zhang K. Wang Z. He J. Lu L. Wang W. Yang A. . (2025). Mechanisms of synovial macrophage polarization in osteoarthritis pathogenesis and their therapeutic implications. Front. Immunol. 16:1637731. doi: 10.3389/fimmu.2025.1637731, 41376619 PMC12685870

[ref166] Zhang B. Xu P. Ablasser A. (2025). Regulation of the cGAS-STING pathway. Annu. Rev. Immunol. 43, 667–692. doi: 10.1146/annurev-immunol-101721-032910, 40085836

[ref167] Zhang K. Zeng L. Min H. Jiang X. Li Y. Hu K. (2026). Crosstalk between programmed cell death in chondrocytes-a molecular mechanism of osteoarthritis. Tissue Cell 98:103161. doi: 10.1016/j.tice.2025.103161, 41027246

[ref168] Zhang R. Zhang L. Tian B. Wang Y. Kang X. Zheng J. (2026). The gut-bone-cartilage triad: microbial regulation of the Wnt/β-catenin signaling pathway in osteoarthritis joint remodeling (review). Mol. Med. Rep. 33:23. doi: 10.3892/mmr.2025.13733, 41170737 PMC12606565

[ref169] Zhao Y. Cheng S. Liang J. Yu G. (2026). The role of tryptophan-AhR signaling in the pathogenesis of metabolic dysfunction-associated steatotic liver disease (MASLD): implications for therapeutic strategies. Front. Immunol. 17:1806118. doi: 10.3389/fimmu.2026.1806118, 41929520 PMC13038574

[ref170] Zhao W. Li J. Su T. Wang C. Fu Y. Li C. . (2025). Osteoclast activation and inflammatory bone diseases: focusing on receptors in osteoclasts. J. Inflamm. Res. 18, 3201–3213. doi: 10.2147/JIR.S507269, 40059949 PMC11890017

[ref171] Zhao C. Lin S. (2025). PANoptosis in intestinal epithelium: its significance in inflammatory bowel disease and a potential novel therapeutic target for natural products. Front. Immunol. 15:1507065. doi: 10.3389/fimmu.2024.1507065, 39840043 PMC11747037

[ref172] Zhao H. Yue N. Mai Z. Zhang Y. Tian C. Kong C. . (2025). *Lactobacillus johnsonii*-derived extracellular vesicles restore mucosal immunity via taurine-linked Th17/Treg and IgA/IgG regulation in colitis. J. Nanobiotechnol. 23:612. doi: 10.1186/s12951-025-03702-6 PMC12482724 41023976

[ref173] Zheng K. Feng Y. Li L. Kong F. Gao J. Kong X. (2024). Engineered bacterial outer membrane vesicles: a versatile bacteria-based weapon against gastrointestinal tumors. Theranostics 14, 761–787. doi: 10.7150/thno.85917, 38169585 PMC10758051

[ref174] Zheng L. Gu M. Li X. Hu X. Chen C. Kang Y. . (2025). ITGA5+ synovial fibroblasts orchestrate proinflammatory niche formation by remodelling the local immune microenvironment in rheumatoid arthritis. Ann. Rheum. Dis. 84, 232–252. doi: 10.1136/ard-2024-225778, 39919897

[ref175] Zheng M. Tian Q. Shen J. Li S. (2026). Dysregulated immunometabolism in gut inflammation. Acta Biochim. Biophys. Sin. Shanghai 58, 169–182. doi: 10.3724/abbs.2025192, 41122068 PMC12862621

